# Diversity of introduced terrestrial flatworms in the Iberian Peninsula: a cautionary tale

**DOI:** 10.7717/peerj.430

**Published:** 2014-06-10

**Authors:** Marta Álvarez-Presas, Eduardo Mateos, Àngels Tudó, Hugh Jones, Marta Riutort

**Affiliations:** 1 Departament de Genètica, Facultat de Biologia i Institut de Recerca de la Biodiversitat (IRBio), Universitat de Barcelona, Barcelona, Spain; 2 Departament de Biologia Animal, Facultat de Biologia, Universitat de Barcelona, Barcelona, Spain; 3 Department of Zoology, The Natural History Museum, London, UK

**Keywords:** Platyhelminthes, Tricladida, Alien species, Habitat restoration, Soil fauna, Molecular identification

## Abstract

Many tropical terrestrial planarians (Platyhelminthes, Geoplanidae) have been introduced around the globe. One of these species is known to cause significant decline in earthworm populations, resulting in a reduction of ecological functions that earthworms provide. Flatworms, additionally, are a potential risk to other species that have the same dietary needs. Hence, the planarian invasion might cause significant economic losses in agriculture and damage to the ecosystem. In the Iberian Peninsula only *Bipalium kewense* Moseley, 1878 had been cited till 2007. From that year on, four more species have been cited, and several reports of the presence of these animals in particular gardens have been received. In the present study we have: (1) analyzed the animals sent by non-specialists and also the presence of terrestrial planarians in plant nurseries and garden centers; (2) identified their species through morphological and phylogenetic molecular analyses, including representatives of their areas of origin; (3) revised their dietary sources and (4) used Species Distribution Modeling (SDM) for one species to evaluate the risk of its introduction to natural areas. The results have shown the presence of at least ten species of alien terrestrial planarians, from all its phylogenetic range. International plant trade is the source of these animals, and many garden centers are acting as reservoirs. Also, landscape restoration to reintroduce autochthonous plants has facilitated their introduction close to natural forests and agricultural fields. In conclusion, there is a need to take measures on plant trade and to have special care in the treatment of restored habitats.

## Introduction

Most animal invasive species detected in Europe are terrestrial invertebrates (Roques et al., 2009). Invading edaphic organisms can have dramatic effects on the environment, due to the direct effects on native soil organisms, and through their interactions with the environment aboveground. However, overall, their impact in human health and economy is greater than their ecological impact (Vilà et al., 2010). Among these organisms, land planarians are becoming an important and diversified group of introduced species in Europe.

**Figure 1:**
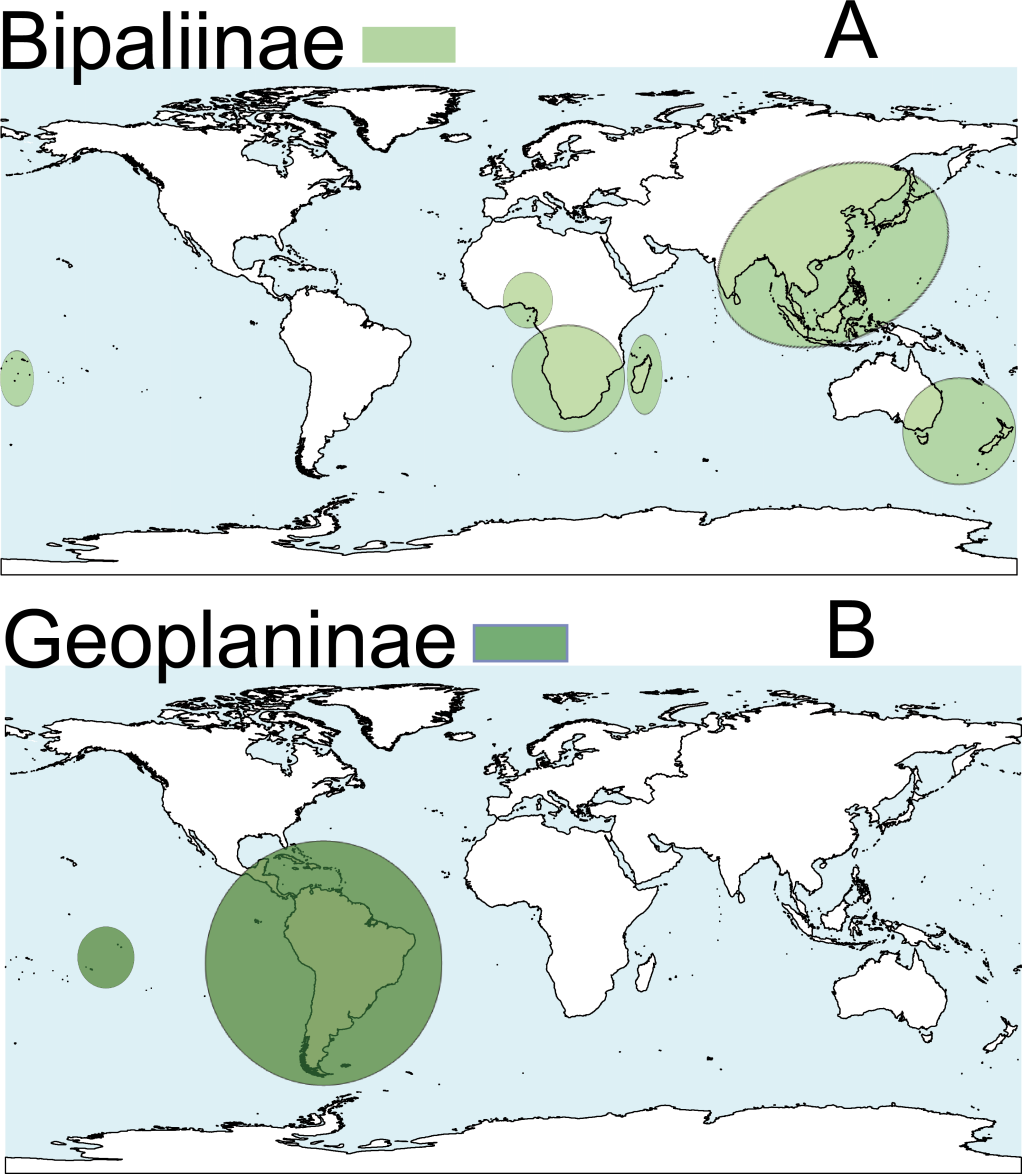
Distribution map of the terrestrial flatworms. (A) Subfamily Bipaliinae. (B) Subfamily Geoplaninae. Information from http://turbellaria.umaine.edu.

**Figure 2:**
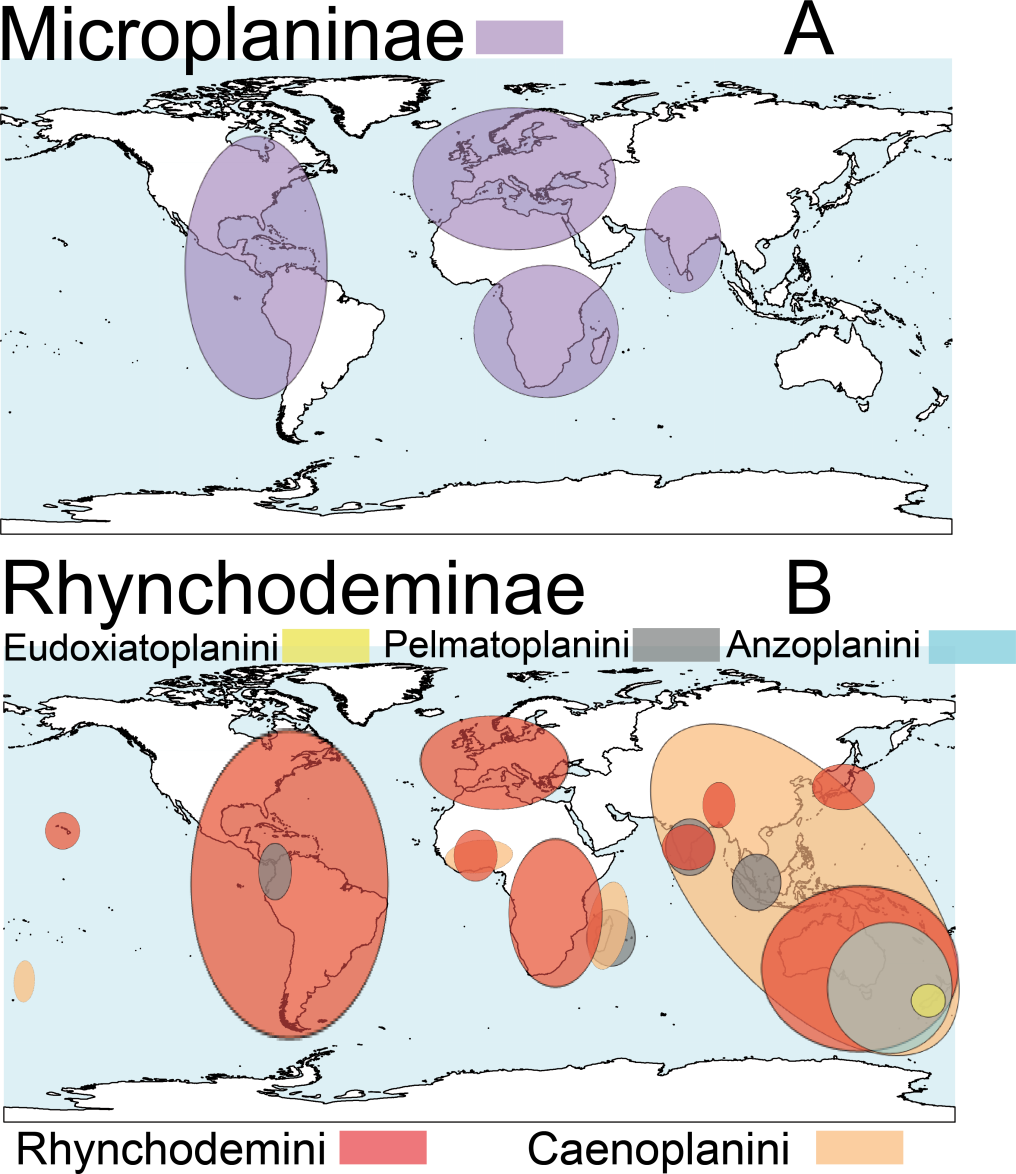
Distribution map of the terrestrial flatworms. (A) Subfamily Microplaninae. (B) Subfamily Rhynchodeminae. Information from http://turbellaria.umaine.edu.

Terrestrial planarians (Platyhelminthes, Geoplanidae) are divided into four subfamilies (Bipaliinae, Microplaninae, Geoplaninae and Rhynchodeminae) with a cosmopolitan distribution (Winsor, Johns & Yeates, 1998); however, most species are found in the southern hemisphere. Bipaliinae (Fig. 1A) is absent from the American and European continents, Geoplaninae (Fig. 1B) have an exclusively Central and South American distribution, while Microplaninae (Fig. 2A) and Rhynchodeminae (Fig. 2B) are the subfamilies with the most northerly distribution, including Europe. Terrestrial planarians are the only free-living Platyhelminthes that do not live in an aquatic habitat. However, they have not developed the capacity to prevent water loss and are thus strongly dependent on environmental moisture levels (Froehlich, 1956; McDonald & Jones, 2007). They seem to withstand this limitation through behavioral strategies such as hiding in damp refuges during the day and becoming active during the night. Due to these characteristics, these animals are considered to have a low capacity to disperse. In fact, in their areas of origin, although a few species are well-adapted to open and human-transformed lands (Baptista & Leal-Zanchet, 2010), most species are restricted to humid forest areas.

A total of 36 species of terrestrial planarians are known to have been introduced in different countries around the globe. Most of these species have a big effect on terrestrial ecosystem processes because they prey on soil invertebrates (see references in Winsor, Johns & Barker, 2004). So far, five of these species are considered to be either invasive and cause problems with local biodiversity (*Platydemus manokwari* De Beauchamp, 1963), or horticultural pests (*Arthurdendyus triangulatus* (Dendy, 1894)) or earthworm farm pests (*Bipalium adventitium* Hyman, 1943; *Bipalium kewense* Moseley, 1878; *Dolichoplana striata* Moseley, 1877, see Winsor, Johns & Barker, 2004).

In Europe there is evidence of the presence of at least 18 introduced terrestrial planarians (Minelli, 1977; Ball & Reynoldson, 1981; Jones, 1988; Jones, 1998; Mateos, Giribet & Carranza, 1998; Faubel, 2004; Jones et al., 2008; Vila-Farré et al., 2008; Vila-Farré et al., 2011; Justine et al., 2014). In the Iberian Peninsula (IP) there are only a few published records of introduced terrestrial planarians, and the only species cited are *Bipalium kewense* in Barcelona (Filella-Subirà, 1983), *Platydemus* sp. in Málaga (Vila-Farré et al., 2011), *Obama* sp. in Asturias (Fernández et al., 2013) and *Rhynchodemus* R02 and *Caenoplana coerulea* Moseley, 1877 in Girona (Mateos et al., 2013). The last species has also been cited in Menorca (Breugelmans et al., 2012).

After receiving multiple reports from non-scientists on the presence of “large and colored” terrestrial flatworms in several localities in the IP, and given their observed locations, particularly in private gardens, we decided to analyze their presence in garden centers and plant nurseries.

The aims of this work were to: (1) estimate the number of terrestrial flatworm species introduced in the IP, and find their region of origin; (2) check whether plant nurseries and garden centers are acting as entrance gates and reservoirs; (3) estimate the invasive potential of some introduced species by considering their diet and by using Species Distribution Modeling (SDM); (4) propose measures to prevent their becoming invasive and to prevent further introductions and spread.

## Material and Methods

### Specimen collection

Specimens were sampled from four sources (Tables 1 and 2): (1) gardens, (2) nurseries and plantations, (3) semi natural areas, and (4) from other countries (either the original area of distribution or other invaded areas). Specimens from sources 1 and 2 were either sent by people who knew our work through the information in social networks, or sampled by us (all the localities reported by non-scientist collaborators correspond to gardens). Specimens from source 3 were sampled by us. Specimens from source 4 were sent by colleagues, specialists of the group, to whom we requested material for comparison with the Iberian populations.

**Table 1:** Localities where introduced species have been found/recorded in the Iberian Peninsula. Data organized chronologically. Sampling code: (fs), specimens from field surveys conducted by us in gardens, nurseries and semi natural areas; (sbp), specimens sent by people who knew our work through the information in social networks; (bd), bibliographic data. Date in format yyyy/mm/dd. Collectors: AG, Alberto Gayoso; AL, Álvaro Leal; AT, Àngels Tudó; CC, Cristina Cabrera; CI, César de Inés; CS, Carmen Soler; EM, Eduardo Mateos; GG, Georgina Gratacós; IV, Iván Salvia; JM, Jacobo Martín; MR, Marta Riutort; RS, Roberto Sáez; VS, Vicent Sancho; Montilivi-WEB, http//www.iesmontilivi.net/WebProfes/jbarbara/web/Galeria/Imatges/Invertebrats/cuc.htm; XB, Xavier Béjar.

Sampling code	Loc code	Locality	Position	Habitat	Species	Date	Collector/Ref
bd	A	Caldes d’Estrac (Barcelona)	N41.569467 E2.526316	garden	*Bipalium kewense*	1983	Filella-Subirà, 1983
fs	B	Barcelona (Barcelona)	N41.398539 E2.142162	garden	*Bipalium kewense*	1995	MR
sbp	C	Lourizan (Pontevedra)	N42.410111 W8.667716	nursery	*Bipalium kewense*	1990	AG
bd	D	Girona (Girona)	N41.964541 E2.827842	garden	*Bipalium kewense*	1994	Montilivi-WEB
sbp	E	Villamalea (Albacete)	N39.362159 W1.601281	nursery	*Bipalium kewense*	1998	VS
sbp	F	Bétera (València)	N39.604153 W0.507864	garden	*Bipalium kewense*	1999	VS
bd	G	Benarmargosa (Málaga)	N36.8248 W4.1809	mango plantation	Rhynchodemini Ri1^G^	2007/12/25	Vila-Farré et al., 2011 as *Platydemus* sp
sbp	H	Badalona (Barcelona)	N41.460177 E2.243985	garden	*Caenoplana* Ca1^G^	2008	RS
bd	I	Menorca (Balearic Islands)	N39.95000 E3.850000	orchard	*Caenoplana coerulea*	2009/04	Breugelmans et al., 2012
fs	J	Torruella de Fluvià (Girona)	N42.17559 E3.03953	garden	*Obama sp.6* ^G^	2010/04/04	MR
sbp	K	Òliva (Valencia)	N38.910550 W0.073200	garden	*Caenoplana* Ca1^G^	2010/11/08	VS
sbp	L	Ames (A Coruña)	N42.857955 W8.653278	garden	*Caenoplana* Ca1	2010/12/10	AG
fs	M	Granollers (Barcelona)	N41.570240 E2.270532	semi natural	*Caenoplana* Ca1 ^G^ *Kontikia ventrolineata*^G^	2011/02/28 2012/10/12	CS EM
sbp	N	Boadilla del Campo (Madrid)	N40.405270 W3.877014	garden	*Caenoplana* Ca1	2011/10/15	JM
fs	O	El Prat de Llobregat (Barcelona)	N41.309519 E2.120887	semi natural	*Caenoplana* Ca1 ^G^^,^^M^	2011/11/05	EM & CC
fs	P	Vall d’en Bas (Girona)	N42.125939 E2.433678	semi natural	*Caenoplana* Ca1 ^G^ *Rhynchodemus* Rs1 ^G^^,^^M^	2011/11/12 2011/11/26	EM & XB EM & MR
fs	Q	Gavà-1 (Barcelona)	N41.288100 E2.006233	nursery	*Obama sp*	2012/03/13	AT & MR
fs	R	Gavà-2 (Barcelona)	N41.293222 E2.017583	nursery	*Obama sp* ^G^	2012/03/14	AT & MR
fs	S	Vilassar de Mar (Barcelona)	N41.497084 E2.376178	nursery	*Obama sp* ^G^	2012/03/28	AT & MR
fs	T	Tortosa (Tarragona)	N40.767329 E0.556963	nursery	*Obama sp* ^G^	2012/04/04	AT
sbp	U	Treto (Cantabria)	N43.392385 W3.470387	garden	*Obama sp*^G^ *Bipalium kewense*^G^	2012/06/27	CI
fs	V	Bordils (Girona)	N42.034804 E2.898615	nursery	*Caenoplana* Ca1^G^ *Caenoplana* Ca2 ^G^^,^^M^ *Caenoplana bicolor*^G^ *Obama sp*^G^ *Dolychoplana striata*^G^ *Bipalium kewense*	2012/10/22	EM
sbp	W	Girona (Griona)	N42.009800 E2.825554	garden	*Caenoplana coerulea*	2013/09/11	GG
sbp	X	Polop (Alicante)	N38.622149 W0.126626	garden	*Caenoplana coerulea*	2014/02/01	AL
sbp	Y	Cártama (Málaga)	N36.748333 W4.586944	garden	*Obama sp*	2014/03/01	IV

**Notes.**

G Species with genetic sequences.

M Species sectioned for internal anatomy study (see Table 2).

**Table 2:** Sequenced specimens. To each new sequence a three digit numeric code was assigned. Sequences from the GenBank database do not have specimen code numbers, only when there are more specimens from the same species in the same locality was a specimen code assigned (three letters + one number). Loc codes are as described in Table 1. Collector: DB, Dani Boix; EM, Eduardo Mateos; HJ, Hugh Jones; KA, Miquel Arnedo; LL, Laia Leria; LW, L Winsor; MA, Marta Álvarez-Presas; SG, S Graham; MV, Miquel Vila.

Species/morphotype	Code	Locality/ref. or Loc code or collector and position	GenBank Code	
			28S	COI
**Family** Geoplanidae				
**Subfamily** Bipaliinae				
*Bipalium* sp.		Japan/Baguñà et al., 2001–Álvarez-Presas, Baguñà & Riutort, 2008	DQ665959 ^a^	AF178307 ^a^^,^^c^
*B. adventitium*		Leignston (USA)/Baguñà et al., 2001–Álvarez-Presas, Baguñà & Riutort, 2008	DQ665956 ^a^	AF178306 ^a^^,^^c^
*B. kewense*	894	Ponta Delgada, Illa São Miguel (Açores, Portugal)/ DB N37.745196 W25.667408^*^	KJ599731 ^a^	KJ659612 ^*^^,^^a^^,^^c^
	621	Treto (Cantabria, Spain)/U^*^	KJ659703 ^*^^,^^a^	KJ659609 ^*^^,^^a^^,^^c^
	623			KJ659610 ^*^^,^^a^^,^^c^
	666	Bordils (Girona, Spain)/V^*^		KJ659611 ^*^^,^^a^^,^^c^
*B. multilineatum*		South Korea /GenBank Direct submission2010-may-18		HM346600 ^c^
*B. nobile*				HM346598 ^c^
*Novibipalium venosum*		Japan/Álvarez-Presas, Baguñà & Riutort, 2008		DQ666048 ^c^
**Subfamily** Microplaninae				
*Microplana nana*		Vall d’en Bas (Girona, Spain)/Mateos et al., 2009	KJ599722 ^a^	FJ969947 ^a^^,^^c^
*M. groga*		Canyamars (Barcelona, Spain)/Mateos et al., 2009	KJ599721 ^a^	FJ969964 ^a^^,^^c^
*M. terrestris*		Pontedeume (A Coruña, Spain)/Mateos et al., 2009	KJ599724 ^a^	FJ969952 ^a^^,^^c^
**Subfamily** Rhynchodeminae				
**Tribe** Caenoplanini				
*Arthurdendyus triangulatus*		Wainui Barrys Bay (NewZealand)/Álvarez-Presas, Baguñà & Riutort, 2008	DQ665953 ^a^	DQ666027 ^a^^,^^b^^,^^d^
*Artioposthia* sp.		Australia/Baguñà et al. 2001–Álvarez-Presas, Baguñà & Riutort, 2008	DQ665954 ^a^	AF178325 ^a^^,^^b^^,^^d^
*A. testacea*		Malborough (Australia)/Baguñà et al. 2001–Álvarez-Presas, Baguñà & Riutort, 2008	DQ665952 ^a^	AF178305 ^a^^,^^b^
*Australoplana* sp.		New Plymouth, Omata (NewZealand)/Álvarez-Presas, Baguñà & Riutort, 2008	DQ665955 ^a^	DQ666028 ^a^^,^^b^^,^^d^
*Caenoplana* sp. 1		-/Álvarez-Presas, Baguñà & Riutort, 2008	DQ665964 ^a^	DQ666031 ^a^^,^^b^^,^^d^
*Caenoplana* sp. 4		-/Álvarez-Presas, Baguñà & Riutort, 2008		DQ666032 ^a^^,^^b^
*Caenoplana bicolor*	654	Bordils (Girona, Spain)/V^*^	KJ659705 ^*^^,^^a^	KJ659648 ^*^^,^^a^^,^^b^
*Caenoplana coerulea s.l.*		New Plymouth, Omata (NewZealand)/Álvarez-Presas, Baguñà & Riutort, 2008	DQ665961 ^a^	DQ666030 ^a^^,^^b^
		Menorca (Balearic islands, Spain)/(Breugelmans et al., 2012)		JQ514564 ^a^^,^^b^
	Tal1	Tallaganda (Australia)/Sunnucks et al., 2006		DQ227621 ^b^
	Tal2			DQ227625 ^b^
	Tal3			DQ227627 ^b^
	Tal4			DQ227629 ^b^
	Tal5			DQ227631 ^b^
	Tal6			DQ227632 ^b^
	Tal7			DQ227633 ^a^^,^^b^
	Tal8			DQ227635 ^a^^,^^b^
	Vic1	Victoria (Australia)/Sunnucks et al., 2006		DQ465372 ^a^^,^^b^
*Caenoplana* morph Ca1	399	El Prat de Llobregat (Barcelona, Spain)/O^*^		KJ659613 ^*^^,^^a^^,^^b^
	400			KJ659614 ^*^^,^^a^^,^^b^
	402			KJ659615 ^*^^,^^b^
	403			KJ659616 ^*^^,^^b^
	404			KJ659617 ^*^^,^^b^
	415	Vall de’n Bas (Girona, Spain)/P^*^		KJ659618 ^*^^,^^a^^,^^b^
	416			KJ659619 ^*^^,^^b^
	417			KJ659620 ^*^^,^^b^
	418			KJ659621 ^*^^,^^b^
	419			KJ659622 ^*^^,^^b^
	420			KJ659623 ^*^^,^^b^
	421			KJ659624 ^*^^,^^b^
	422			KJ659625 ^*^^,^^b^
	423			KJ659626 ^*^^,^^b^
	424			KJ659627 ^*^^,^^b^
	443	Badalona (Barcelona, Spain)/H^*^	KJ659700 ^*^^,^^a^	KJ659633 ^*^^,^^a^^,^^b^
	444			KJ659634 ^*^^,^^a^^,^^b^
	445			KJ659635 ^*^^,^^b^
	446			KJ659636 ^*^^,^^b^
	450			KJ659637 ^*^^,^^b^
	451			KJ659638 ^*^^,^^b^
	452			KJ659639 ^*^^,^^b^
	453			KJ659640 ^*^^,^^b^
	454	Òliva (València, Spain)/K^*^		KJ659641 ^*^^,^^a^^,^^b^
	601	Garden, Adelaide (Australia)/SG S34.988611 E138.599722^*^	KJ659702 ^*^^,^^a^	KJ659642 ^*^^,^^a^^,^^b^
	603	Garden in Townsville (Palmetum, Australia)/ LW S19.260277 E146.822222^*^		KJ659643 ^*^^,^^a^^,^^b^
	605			KJ659644 ^*^^,^^a^^,^^b^
	634	Nursery in Liverpool (UK)/HJ N53.3525 W2.902777^*^		KJ659645 ^*^^,^^a^^,^^b^
	649	Granollers (Barcelona, Spain)/M^*^		KJ659646 ^*^^,^^a^^,^^b^
	650			KJ659647 ^*^^,^^a^^,^^b^
	735	El Prat de Llobregat (Barcelona, Spain)/O^*^		KJ659651 ^*^^,^^a^^,^^b^
*Caenoplana* morph Ca2	426	Bordils (Girona, Spain)/V^*^		KJ659628 ^*^^,^^b^
	427			KJ659629 ^*^^,^^b^
	428			KJ659630 ^*^^,^^b^
	430		KJ659699 ^*^^,^^a^	KJ659631 ^*^^,^^a^^,^^b^
	431			KJ659632 ^*^^,^^a^^,^^b^
	657			KJ659649 ^*^^,^^a^^,^^b^
	658			KJ659650 ^*^^,^^a^^,^^b^
**Tribe** Rhynchodemini				
*Dolichoplana sp.*		-/Álvarez-Presas, Baguñà & Riutort, 2008	DQ665971 ^a^	DQ666037 ^a^^,^^b^^,^^d^
*D. striata*		Igreginha (Brazil)/Carbayo et al., 2013	KC608341 ^a^	KC608226 ^a^^,^^b^^,^^d^
	425	Bordils (Girona, Spain)/V^*^	KJ659698 ^*^^,^^a^	KJ659679 ^*^^,^^a^^,^^d^
	660			KJ659683 ^*^^,^^a^^,^^d^
	661			KJ659684 ^*^^,^^a^^,^^d^
*Kontikia ventrolineata*	638	Granollers (Barcelona, Spain)/M^*^		KJ659681 ^*^^,^^a^^,^^d^
	639		KJ659704 ^*^^,^^a^	KJ659682 ^*^^,^^a^^,^^d^
	734	Nursery in Liverpool (UK)/HJ N53.3525 W2.902777^*^		KJ659687 ^*^^,^^a^^,^^d^
	739	Saint Pée sur Nivelle (France)/MA N43.34235 W1.52650^*^	KJ599732 ^a^	KJ659688 ^*^^,^^a^^,^^d^
*Platydemus manokwari*		Townsville (Australia)/Baguñà et al., 2001- Álvarez-Presas, Baguñà & Riutort, 2008	DQ665986 ^a^	AF178320 ^a^^,^^b^ ^d^
*Rhynchodemus* morph Rs1	411	Vall de’n Bas (Girona, Spain)/P^*^	KJ659697 ^*^^,^^a^	KJ659676 ^*^^,^^a^^,^^d^
	412			KJ659677 ^*^^,^^a^^,^^d^
	414			KJ659678 ^*^^,^^a^^,^^d^
	908	Luarca, Asturias (Spain)/LL 43°32′30.81″N6 °32′7.42″O^*^		KJ659696 ^*^^,^^d^
*Rhynchodemus* cf. *sylvaticus*		Canyamars (Barcelona, Spain)/Mateos et al., 2009		FJ969946 ^a^^,^^b^ ^d^
	091	Canyamars (Barcelona, Spain)/EM N41.598317 E2.44302^*^		KJ659672 ^*^^,^^d^
	219	Montjuïc (Barcelona, Spain)/Vila-Farré et al., 2008^*^		KJ659673 ^*^^,^^d^
	569	Underbarrow (UK)/N54.31776 W2.80783^*^		KJ659680 ^*^^,^^d^
	670	Pont en Royans (France)/N45.037875 E5.377033^*^		KJ659685 ^*^^,^^d^
	675		KJ659706 ^*^^,^^a^	KJ659686 ^*^^,^^d^
	905	Benamargosa (Málaga, Spain)/Vila-Farré et al., 2011^*^		KJ659694 ^*^^,^^d^
	900	Sueiro, Asturias (Spain)/LL N43.527130 W6.877329^*^		KJ659689 ^*^^,^^d^
	901	Aljezur (Portugal)/LL N 37.316146 W8.803392^*^		KJ659690 ^*^^,^^d^
	906	Benamargosa (Málaga, Spain)/Vila-Farré et al., 2011^*^		KJ659695 ^*^^,^^d^
Rhynchodemini morph Ri1	902	Benamargosa (Málaga, Spain)/Vila-Farré et al., 2011^*^		KJ659691 ^*^^,^^d^
	903	Benamargosa (Málaga, Spain)/Vila-Farré et al., 2011^*^		KJ659692 ^*^^,^^d^
	904	Benamargosa (Málaga, Spain)/Vila-Farré et al., 2011^*^		KJ659693 ^*^^,^^d^
Rhynchodemini sp2	262	Int. Park la Amistad, Pila (Panamá)/KA N8.524944 W82.618777^*^		KJ659674 ^*^^,^^d^
Rhynchodemini sp3	264			KJ659675 ^*^^,^^d^
**Subfamily** Geoplaninae				
*Cratera crioula*		São Paulo (Brazil)/Carbayo et al., 2013	KC608441 ^a^	KC608324 ^a^^,^^e^
*C. tamoia*		Teresópolis (Brazil)/Carbayo et al., 2013	KC608369 ^a^	KC608254 ^a^^,^^e^
*Obama* sp.6	Bra1	Paulo Lopes (Brazil)/Carbayo et al., 2013	KC608425 ^a^	KC608308 ^a^^,^^e^
	Bra2		KC608426 ^a^	KC608309 ^a^^,^^e^
*Obama* sp.	434	Bordils (Girona, Spain)/V^*^		KJ659652 ^*^^,^^a^^,^^e^
	437			KJ659653 ^*^^,^^a^^,^^e^
	438			KJ659654 ^*^^,^^a^^,^^e^
	667			KJ659663 ^*^^,^^a^^,^^e^
	668			KJ659664 ^*^^,^^a^^,^^e^
	458	Gavà (Barcelona, Spain)/Q^*^		KJ659655 ^*^^,^^a^^,^^e^
	459			KJ659656 ^*^^,^^a^^,^^e^
	593	Vilassar de Mar (Barcelona, Spain)/S^*^		KJ659657 ^*^^,^^a^^,^^e^
	594			KJ659658 ^*^^,^^a^^,^^e^
	595		KJ659701 ^*^^,^^a^	KJ659659 ^*^^,^^a^^,^^e^
	596	La Sínia (Tarragona, Spain)/T^*^		KJ659660 ^*^^,^^a^^,^^e^
	610	Treto (Cantabria, Spain)/U^*^		KJ659661 ^*^^,^^a^^,^^e^
	611			KJ659662 ^*^^,^^a^^,^^e^
	895	Nursery in Liverpool (UK)/HJ N53.3525 W2.902777^*^		KJ659667 ^*^^,^^a^^,^^e^
	896			KJ659668 ^*^^,^^a^^,^^e^
	897			KJ659669 ^*^^,^^a^^,^^e^
	898			KJ659670 ^*^^,^^a^^,^^e^
	754	Torruella de Fluvià (Girona, Spain)/J^*^		KJ659665 ^*^^,^^a^^,^^e^
	755			KJ659666 ^*^^,^^a^^,^^e^
	899			KJ659671 ^*^^,^^a^^,^^e^
*O. burmeisteri*		São Paulo (Brazil)/Álvarez-Presas et al., 2011-Carbayo et al., 2013	KC608349 ^a^	HQ542895 ^a^^,^^e^
*O. josefi*		São Francisco de Paula (Brazil)/Carbayo et al., 2013	KC608435 ^a^	KC608318 ^a^^,^^e^
*O. ladislavii* sensu Froehlich, 1959	Bra1	Blumenau (Brazil)/Carbayo et al., 2013	KC608371 ^a^	KC608256 ^a^^,^^e^
*O. ladislavii* von Graff, 1899	Bra2		KC608373 ^a^	KC608258 ^a^^,^^e^
**OUTGROUP: Family** Dugesiidae				
*Dugesia gonocephala*		The Netherlands/Álvarez-Presas, Baguñà & Riutort, 2008	DQ665965 ^a^	DQ666033 ^a^
*D. ryukyuensis*		-/Baguñà et al., 2001-Álvarez-Presas, Baguñà & Riutort, 2008	DQ665968 ^a^	AF178311 ^a^
*D. subtentaculata*		Spain/Álvarez-Presas, Baguñà & Riutort, 2008	DQ665970 ^a^	DQ666036 ^a^

**Notes.**

a Concatenated dataset.

b Caenoplanini dataset.

c Bipaliinae dataset.

d Rhynchodemini dataset.

e Geoplaninae dataset.

* Sequences obtained in this study.

**Figure 3:**
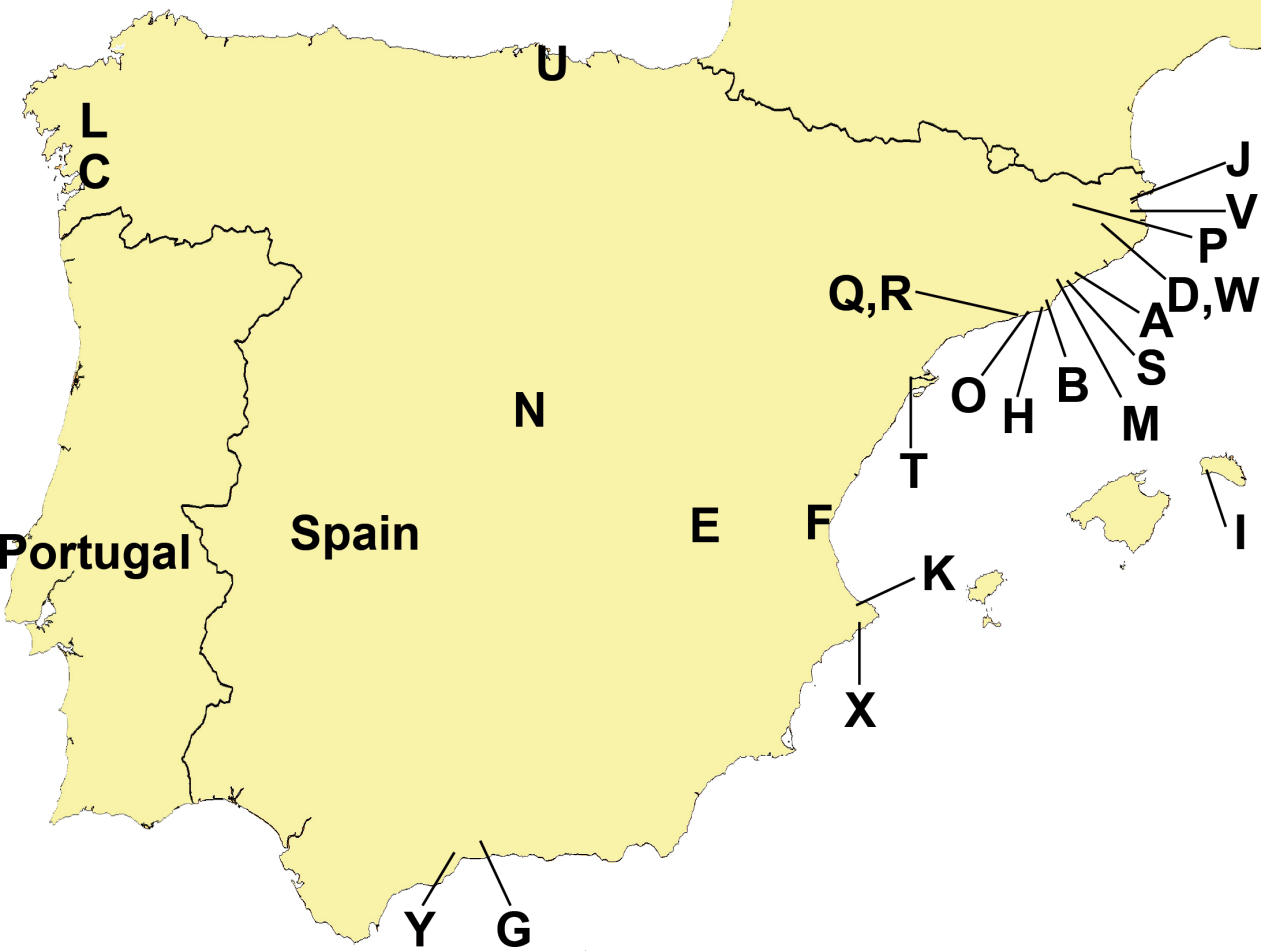
Distribution of sampling localities of introduced terrestrial flatworms in the Iberian Peninsula. Locality codes correspond to those in Table 1.

Data from a total of 13 domestic gardens, seven nurseries, two plantations (all confined, humanized locations), and three semi natural areas (humanized environments that are not confined and in direct contact with agricultural and forest areas) have been analyzed (Table 1). The three “semi natural areas”, located in North-eastern Iberian Peninsula, were: (1) Cal Tet, Parc Natural Delta del Llobregat, Barcelona (Fig. 3, Loc-code O); (2) Can Cabanyes, Granollers, Barcelona (Fig. 3, Loc-code M); (3) Viaducte de Rubiò, Vall d’en Bas, Girona (Fig. 3, Loc-code P). In all three places recent habitat restoration activities have been performed, including the transplantation of autochthonous plant species from commercial nurseries.

Amateur collaborators photographed the animals alive and fixed them in absolute ethanol. Specimens we collected were also photographed and external morphological characters recorded. Subsequently, animals were subjected to two different procedures to proceed to the species identification: (1) specimens for molecular analyses were fixed in 100% ethanol and (2) specimens for histological studies were killed with boiling water, fixed with 10% formalin for 24 h, and then preserved in 70% ethanol.

### Morphological studies

Preserved specimens were examined under a stereo microscope and notes of their dimensions, appearance, color (though this is affected by preservation), eyes, any stripes or pattern, the position of the pharyngeal aperture (mouth) and gonopore, if present, were taken. Specimens with no visible gonopore were considered to be immature. It was possible to identify some specimens, even immature ones, to species level without further examination. For unrecognized specimens, or where identity was uncertain and required confirmation, a mature specimen (evidenced by an open gonopore) was selected and divided into various portions, being embedded in wax. The copulatory apparatus (gonopore) and a small anterior region were sagittally and transversely sectioned at 10 or 15 µm, respectively, stained in Harris’ haematoxylin and eosin and mounted in Canada balsam.

### DNA extraction, gene amplification and sequencing

A small piece of tissue fixed in absolute alcohol was digested with Wizard Genomic DNA Purification lysis Buffer (Promega, Madison, WI, USA) and Proteinase K overnight at 37 °C, following manufacturer’s instructions. The rest of the tissue is kept as voucher in the Genetics Department (Universitat de Barcelona).

We amplified an approximately 1 kb fragment of the mitochondrial cytochrome c oxidase I (Cox1 gene) and a fragment of approximately 1,500 bp of the 28S rRNA gene (28S) by PCR reaction. PCRs were carried out in a volume reaction mixture of 25 µl. For Cox1 we used primers BarS (Álvarez-Presas et al., 2011) and COIR (Lázaro et al., 2009) and conditions were as in Álvarez-Presas et al. (2011); 28S rDNA gene was amplified in two different overlapping fragments using the primers 28S1F, 28S4R, 28S2F and 28S6R, and conditions as in Álvarez-Presas, Baguñà & Riutort (2008). Amplification products were purified with a vacuum manifold (Multiscreen_HTS Vacuum Manifold; Millipore Corporation, Billerica, MA, USA). DNA sequences were determined from both strands using Big-Dye Terminator (3.1, Applied Biosystems, Foster City, CA, USA) and the reaction products were separated on the ABI Prism 3730 automated sequencer (Unitat de Genòmica dels Centres Científics i Tecnològics de la UB).

PCR products of the 28S gene for some individuals, that yielded double bands in the direct sequences, were cloned using HTP TOPO TA Cloning Kit for Sequencing (Invitrogen) in order to be sure that only one type of sequence was recovered (since the existence of a duplication of the ribosomal cluster is known in terrestrial planarians, Carranza et al., 1996). The sequences of the clones showed that these bands corresponded to polymorphisms of one of the types. Seqman (v. 4.2.2, Gene Codes) was used to revise the chromatograms and obtain the definitive sequences.

### Molecular data analyses

Ribosomal sequences were aligned using MAFFT v. 7 (Katoh & Standley, 2013) with the G-INS-i iterative refinement method and 1000 cycles. Mitochondrial coding DNA sequences were translated into aminoacids and aligned manually in Bioedit v.7.0.9.0. (Hall, 1999). All sequences were unambiguously aligned. We estimated the DNA sequence evolution model that best fits the data for both molecules using jModelTest 2.1.4. (Darriba et al., 2012), applying the Akaike information criterion (AIC). Phylogenetic relationships were estimated by Maximum Likelihood (ML) using RAxML 7.0.0 software (Stamatakis, 2006) and Bayesian inference (BI) using MrBayes v. 3.2. (Ronquist et al., 2012). Bootstrap support (BS) values were obtained for ML trees from 10,000 replicates. In the BI analyses we ran four chains to allow heating and used default priors, three million generations were run using the Markov Chain Monte Carlo (MCMC) analysis in two independent runs. Sampling was every 1,000 generations. The stationarity and convergence of the runs were checked by plotting Log likelihood values vs. number of generations and inspecting when the standard deviation of split frequencies had reached <0.01, respectively.

### Potential distribution modeling

Using data describing the known distribution of *C. coerulea* in Australia, we estimated the potential distribution of this species in the Iberian Peninsula, as an exercise to find out whether climatic variables could detect potentially at risk areas where the establishment of the introduced species will be favored if only affected by climate. This could be a tool to help limit potential activities in order to avoid the introduced animals becoming invasive in the most likely areas for them to be successful.

For the SDM, a total of 179 Australian geographical coordinates of presence observations extracted from the literature, internet sources and personal communications (L Winsor, 2013) were used for calibration of models (training dataset). To avoid over-parameterization and loss of predictive power, we discarded the climatic variables that were highly correlated. To do this we extracted environmental information from 10,000 randomly generated points and determined the linear relationships among them using Spearman and Pearson correlations. Although all correlations were significant they show low correlation coefficients (*r* ≤ 0.12). According to this analysis we used the 9 bioclimatic variables from the WorldClim database v. 1.4. (http://www.worldclim.org/, Hijmans et al., 2005) with less dependence, to form the present climatic dataset at a scale of 30 arc s. Those variables were: annual mean temperature; mean diurnal range; isothermality; maximum temperature of warmest month; minimum temperature of coldest month; precipitation of wettest month; precipitation seasonality; precipitation of wettest quarter; and precipitation of warmest quarter. The maximum entropy model, a presence-only algorithm that requires known species occurrence points and environmental variables (Maxent v.3.3.3k; Phillips, Anderson & Schapire, 2006), was applied. We selected the software default values for the convergence threshold, regularization values, and features. The maximum number of iterations was set to 1,000 and 1,000 bootstrap replicates were used. All possible geographic locations were partitioned between training and test samples (75% and 25%, respectively) in order to achieve higher predictive accuracy (Phillips & Dudík, 2008). Once the models were trained, we projected the results using the IP climatic dataset, to study the possible expansion of *C. coerulea* in the region. Model performance was evaluated using the AUC test (area under the receiver operating characteristic curve (ROC)) and the binomial test of the omission-dependent threshold was calculated by Maxent. Finally, binary maps of the outcome of the models were overlapped in the geographic information system, ArcMap v.10 (ESRI 2011. ArcGIS Desktop: Release 10. Redlands, CA: Environmental Systems Research Institute).

## Results

### Morphological identification of the specimens

Based on the external appearance of the flatworms we initially grouped the specimens into nine morphotypes. We classified four of them at the species level due to their characteristic shapes or other external features, and the other five at genus or tribe level.

**Figure 4:**
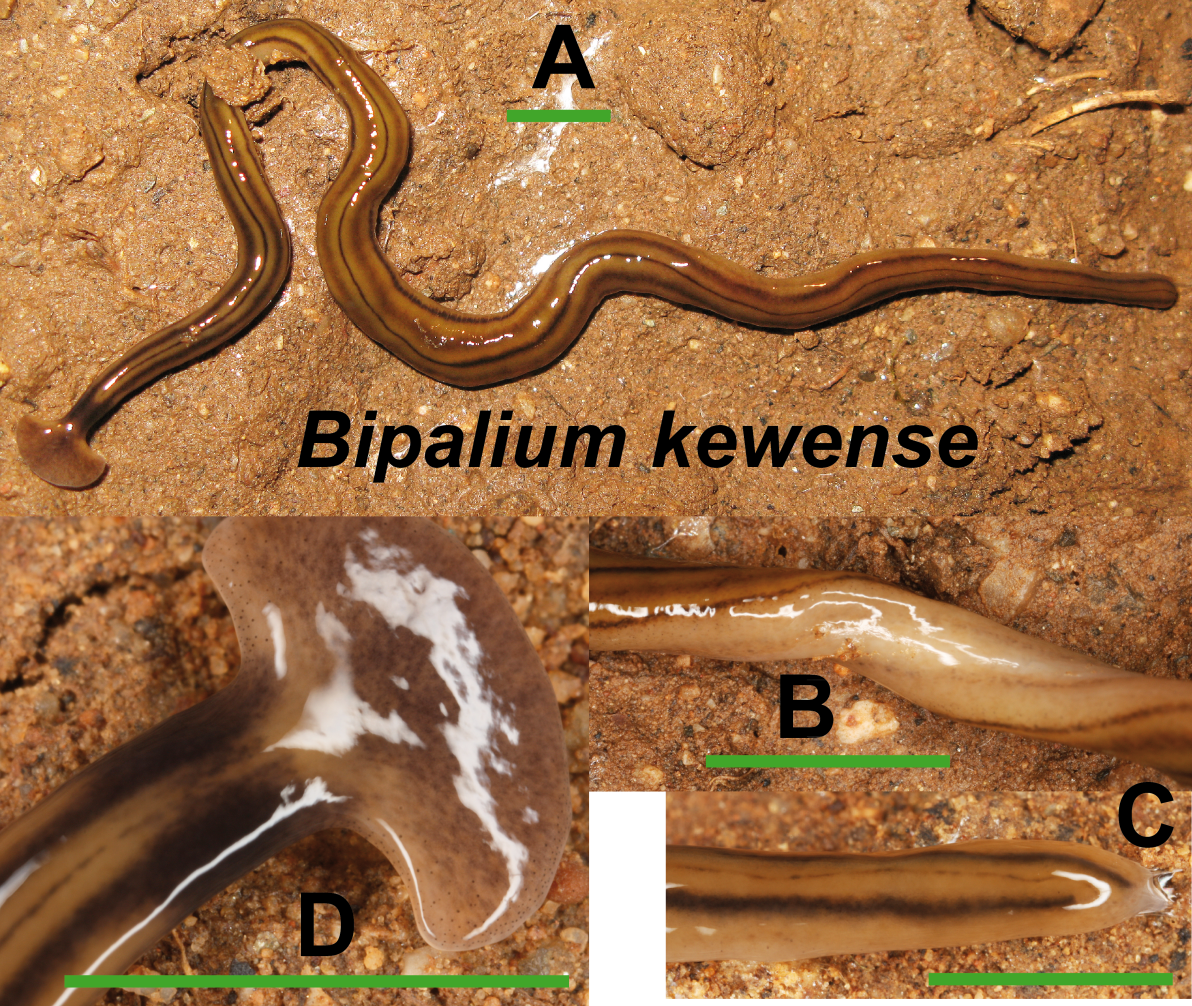
*Bipalium kewense*. (A) Dorsal view. (B) Ventral view of median part. (C) Dorsal view of posterior end. (D) Dorsal view of anterior end. Scale bar 5 mm.

*Bipalium kewense* (Fig. 4) has been identified by the characteristic shape of the anterior end and the pattern of stripes along the dorsal and ventral body surfaces. One specimen preserved in 70% ethanol from Bordils locality (Loc code V in Table 1) has been deposited at the Natural History Museum of the United Kingdom (NHMUK) with voucher number **NHMUK 2014.5.13.6.**

**Figure 5:**
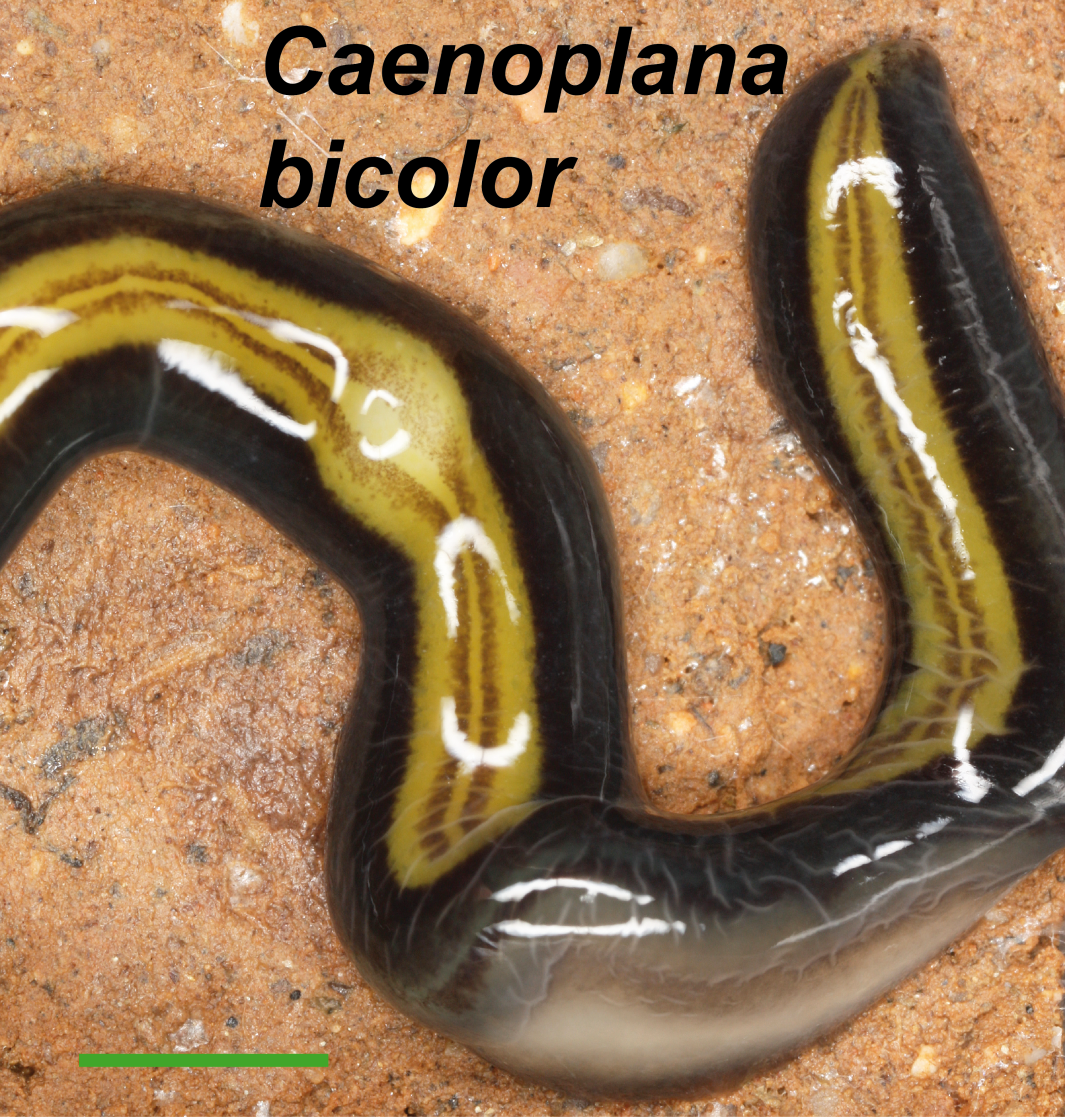
*Caenoplana bicolor*. Dorsal view with partial ventral view in the center. The anterior end is not shown (the specimen was damaged in this region). Scale bar 5 mm.

For *Caenoplana bicolor* (Graff, 1899) there is no published description of a sexually mature specimen, hence the identification of the only specimen obtained, also an immature individual, relied exclusively on its external appearance (Fig. 5). This specimen is deposited in the tissue collection of the Genetics Department (Universitat de Barcelona).

**Figure 6:**
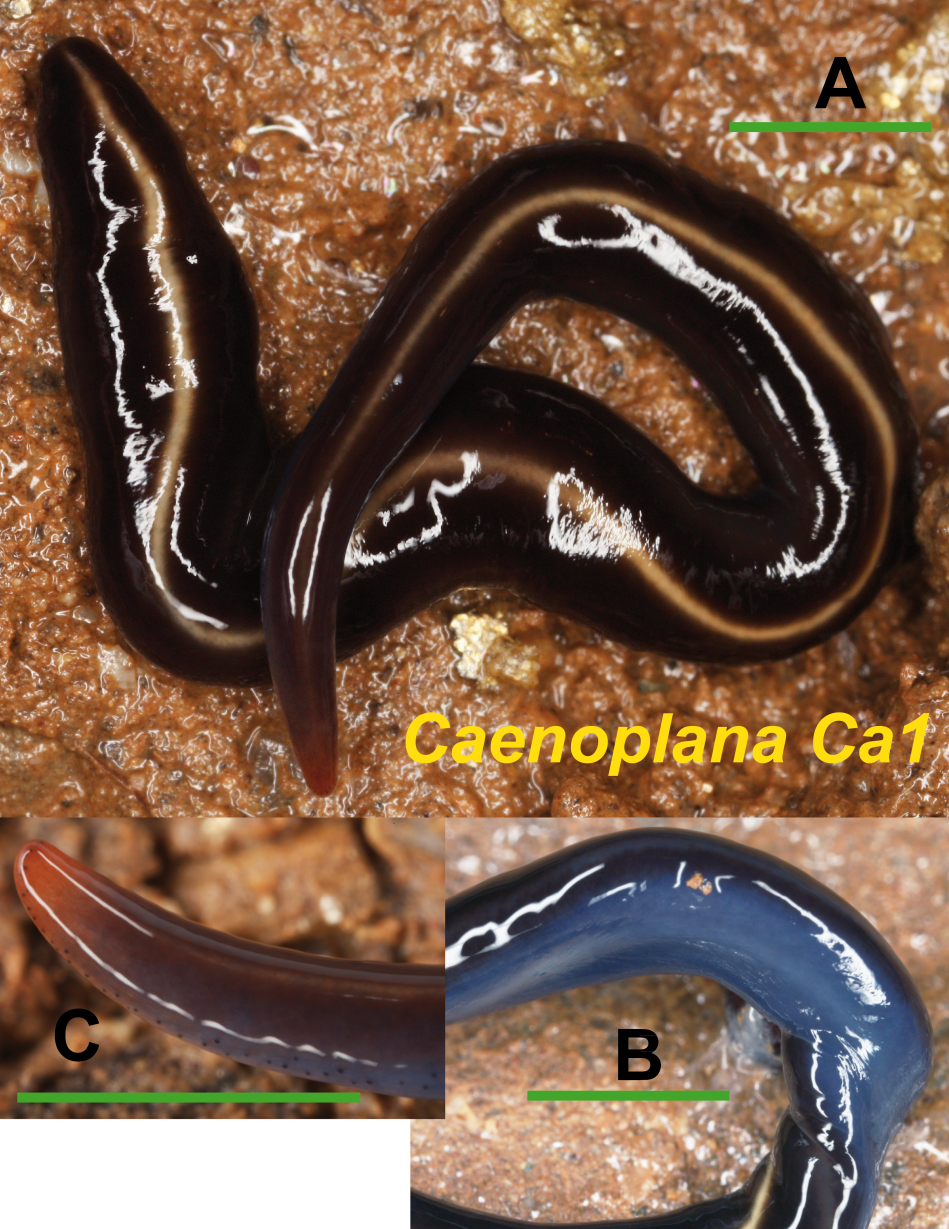
*Caenoplana* morph Ca1. (A) Dorsal view. (B) Ventral view of median part. (C) Lateral view of anterior end showing line of eyes. Scale bar 5 mm.

**Figure 7:**
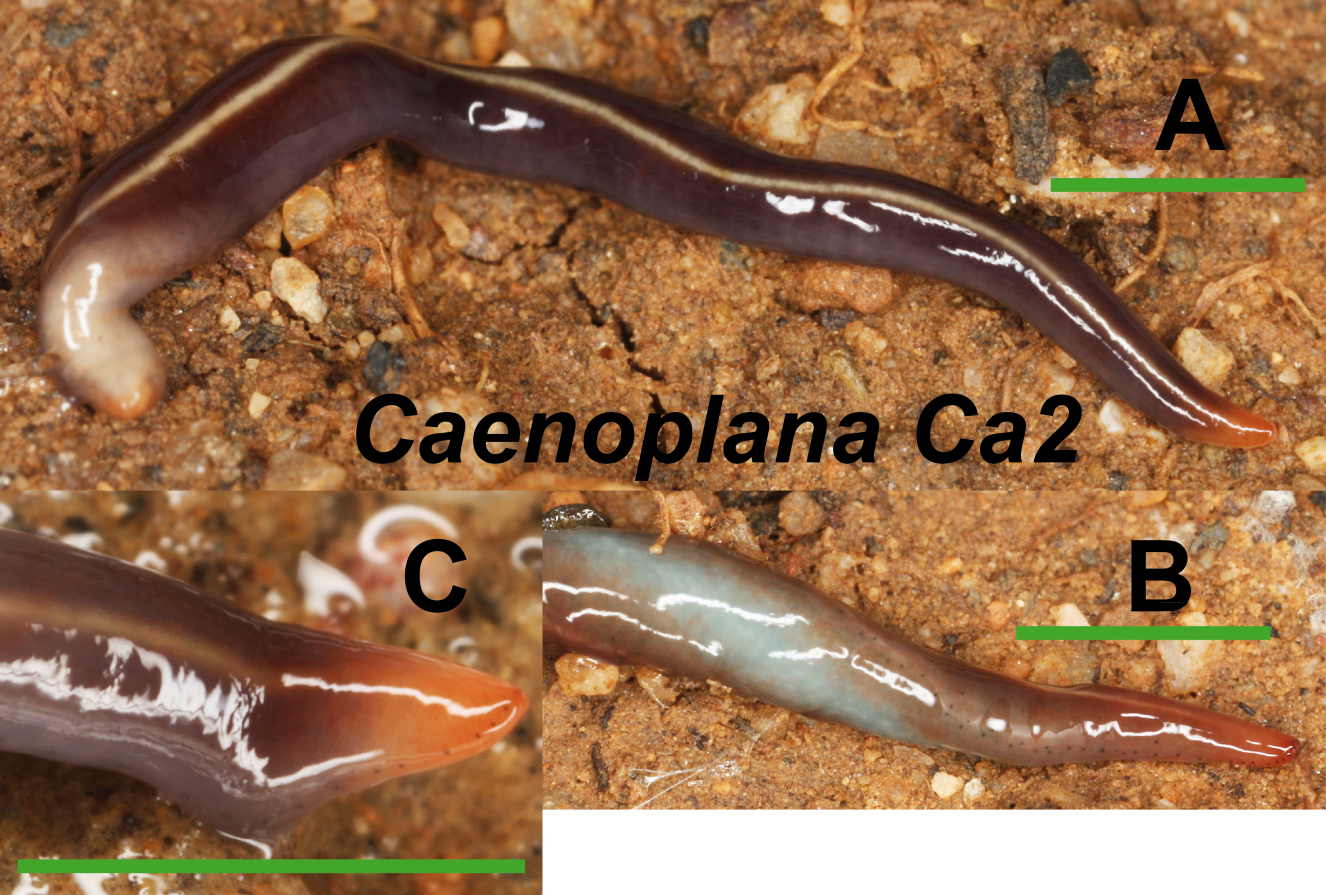
*Caenoplana* morph Ca2. (A) Dorsal view. (B) Ventral view of median part and dorsal view of anterior end showing line of eyes. (C) Lateral view of anterior end showing line of eyes. Scale bar 5 mm.

Among the specimens with an external morphology initially ascribable to the *Caenoplana coerulea* phenotype, we have found two morphotypes basing on their color pattern. Morphotype Ca1 (Fig. 6) presents a dorsal coloration in dark blue with a yellow middle-dorsal stripe, and a ventral light blue region (characteristic pattern of *Caenoplana coerulea*). The histological study of one specimen from El Prat de Llobregat locality (Loc code O in Table 1) (**NHMUK 2014.5.13.14**) reveals that it may belong to the *Caenoplana coerulea* species. Morphotype Ca2 (Fig. 7) presents a light brown dorsal region with a pale yellow middle-dorsal stripe, and a ventral light blue-greenish region. The histological study of one specimen from Bordils locality (Loc code V in Table 1) (**NHMUK 2014.5.13.12**) has revealed that its copulatory apparatus characters do not fit any of the described *Caenoplana* species.

**Figure 8:**
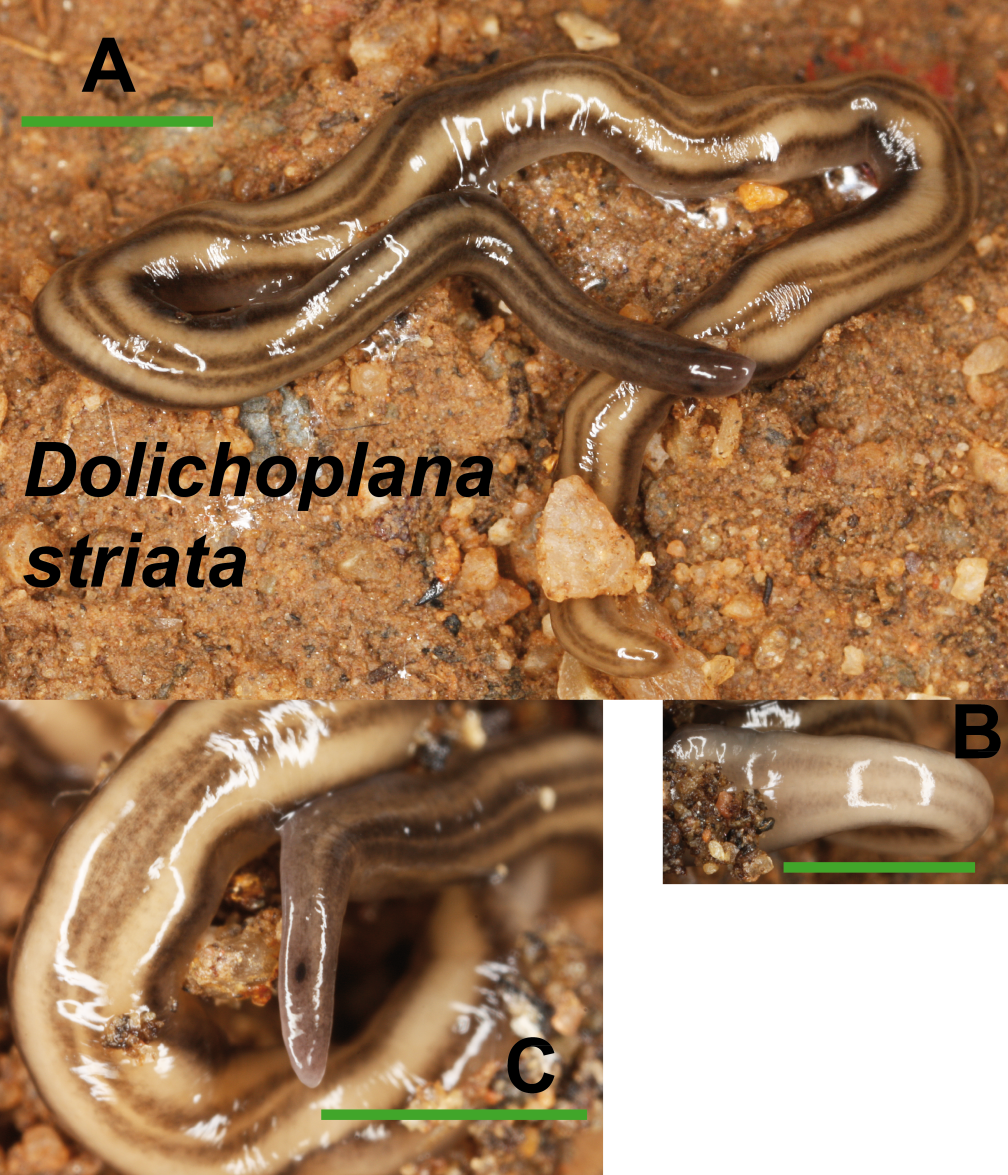
*Dolichoplana striata*. (A) Dorsal view. (B) Ventral view of median part. (C) Lateral view of anterior end showing the eye spot. Scale bar 5 mm.

*Dolichoplana striata* (Fig. 8) could also be identified by its characteristic external appearance. One specimen from Bordils locality (Loc code V in Table 1) has been deposited at the NHMUK (**NHMUK 2014.5.13.7**).

**Figure 9:**
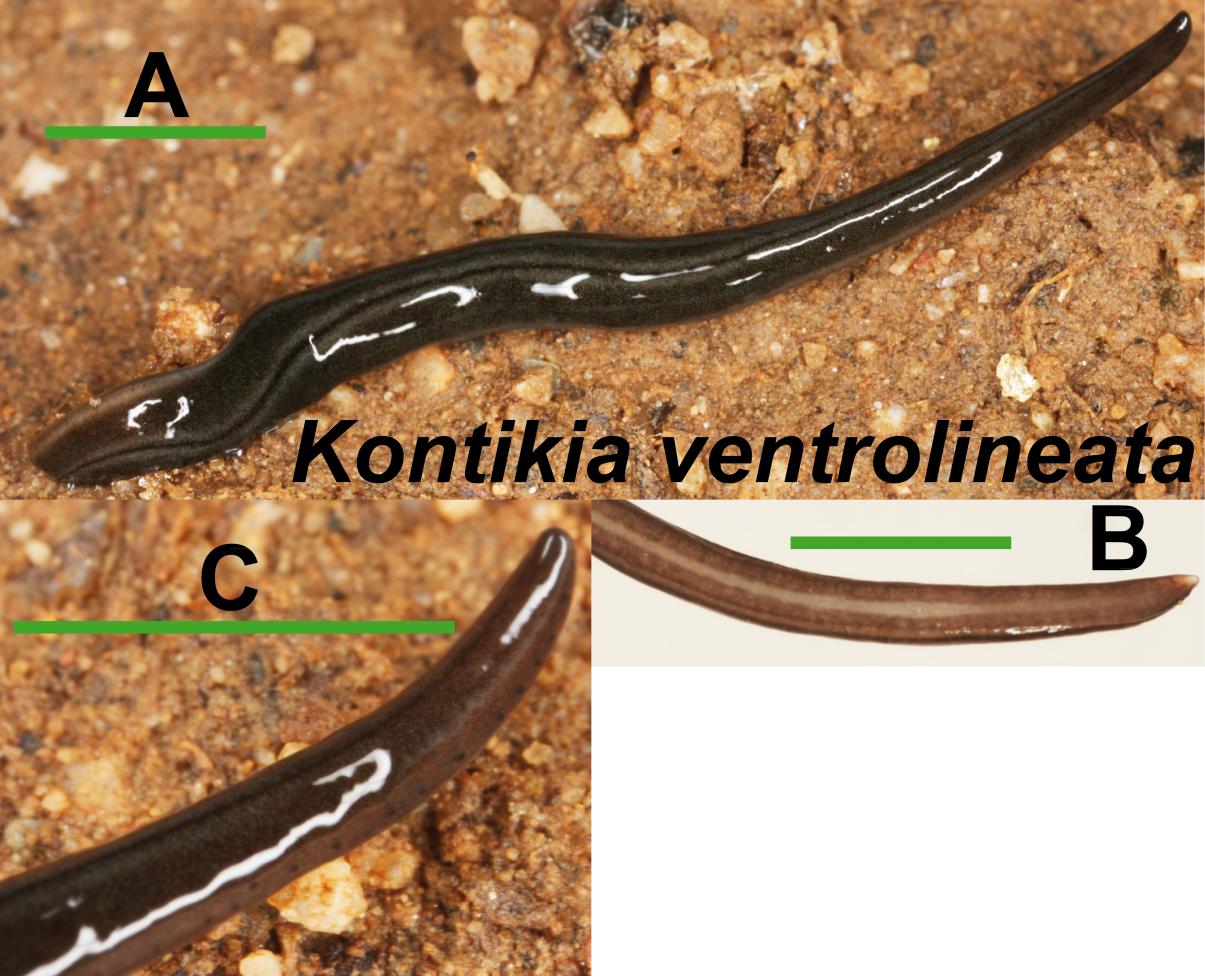
*Kontikia ventrolineata*. (A) Dorsal view. (B) Ventral view of posterior end. (C) Lateral view of anterior end showing line of eyes. Scale bar 5 mm.

*Kontikia ventrolineata* (Dendy, 1892) (Fig. 9) was externally identified, following Great Britain Non-Native Species Secretariat (2013). We assigned the specific name following Jones, Johns & Winsor (1998), who considered *Parakontikia* Winsor, 1991 as a junior synonym of *Kontikia* Froehlich, 1955. Three specimens from Granollers locality (Loc code M in Table 1) are deposited at the NHMUK (**NHMUK 2014.5.13.3-5**).

**Figure 10:**
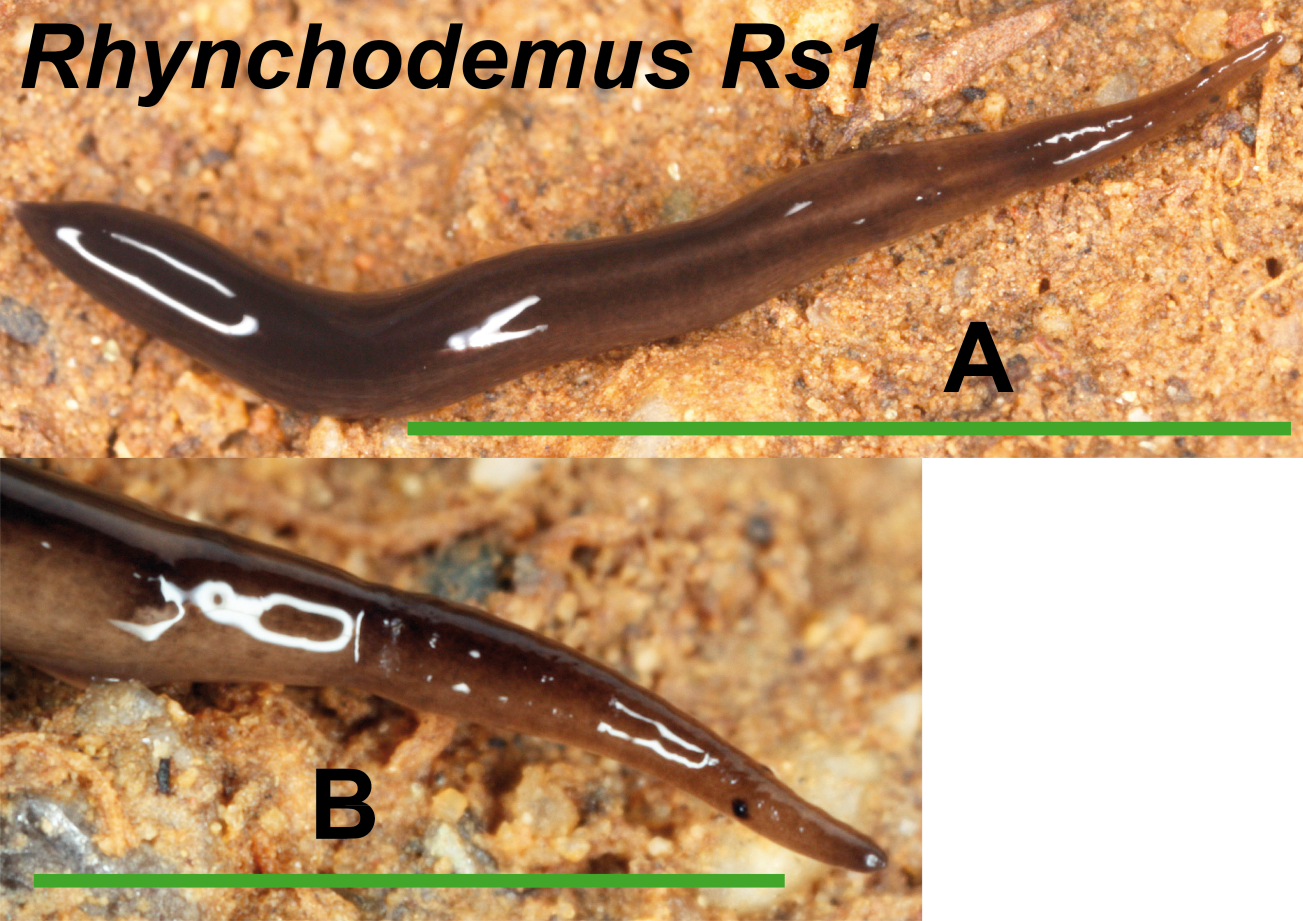
*Rhynchodemus* morph Rs1. (A) Dorsal view, scale bar 5 mm. (B) Lateral view of anterior region, scale bar 2.5 mm.

We found one morphotype externally ascribable to the genus *Rhynchodemus*, but not to a known species (Fig. 10). *Rhynchodemus* morph Rs1 has a dark brown pigmented body with two black longitudinal stripes, and two large eyes situated a little distant from the anterior tip. One specimen from Vall d’en Bas locality (Loc code P in Table 1) (**NHMUK 2014.5.13.9**) was histologically studied but, unluckily, presented a copulatory apparatus not well developed, preventing us from determining whether it could belong to *Rhynchodemus sylvaticus* (Leidy, 1851) to which it was extremely externally similar.

A morphotype externally ascribable to the tribe Rhynchodemini was found in Benamargosa locality (Loc-code G), but its morphological features did not allow assigning it to any genus. Rhynchodemini morph Ri1 presents a dark brown pigmented body with one dorsal black line (no image available).

**Figure 11:**
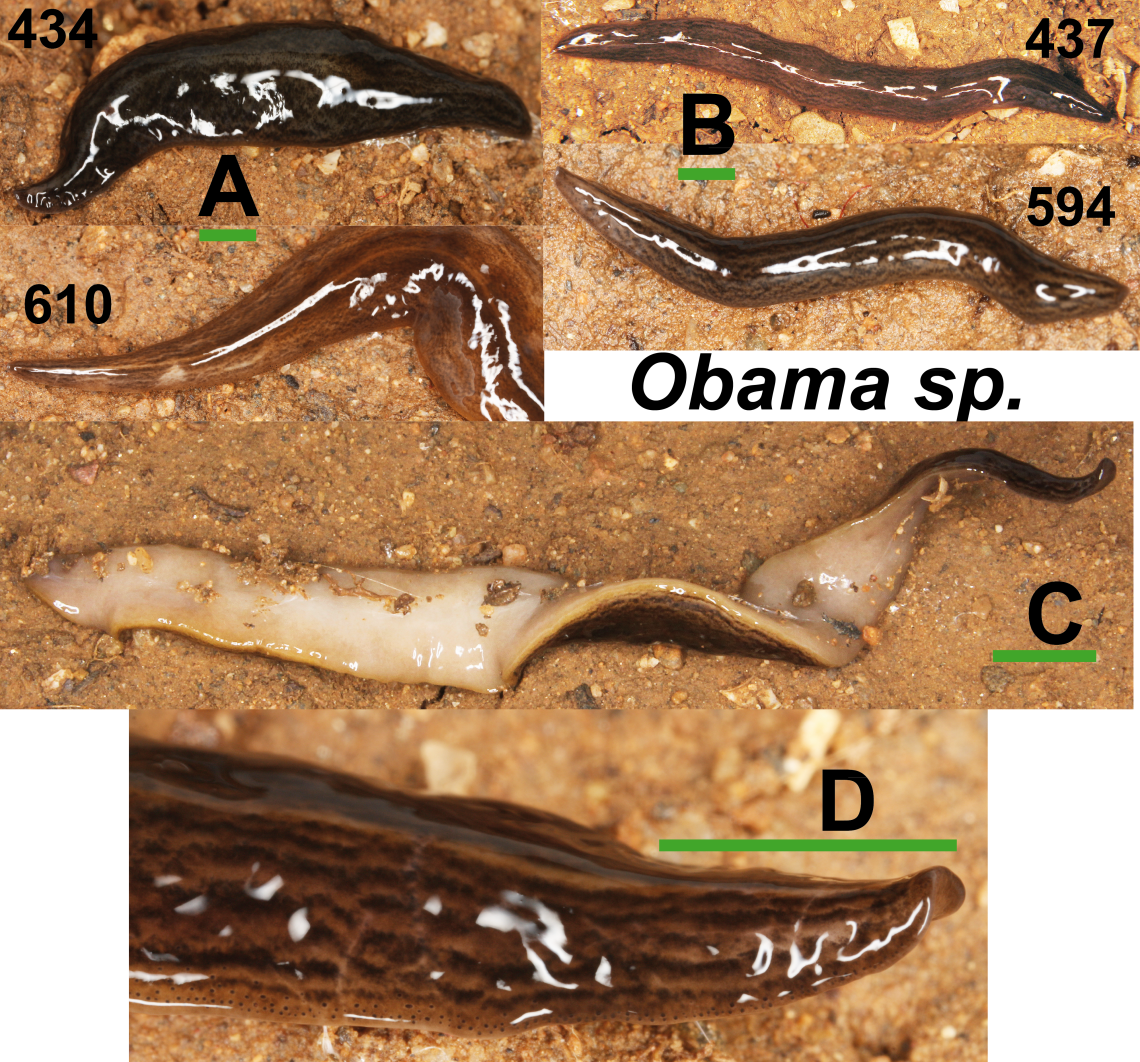
*Obama* sp. (A) Dorsal view of two specimens (codes 434 and 610) from one *Obama* sp. A clade in the Geoplaninae Cox1 tree (Fig. 14). (B) Dorsal view of two specimens (codes 437 and 594) from *Obama* sp. B clade in the Geoplaninae Cox1 tree (Fig. 14). (C) Ventral view. (D) Lateral view of anterior end showing line of eyes. Scale bar 5 mm.

Specimens of *Obama* sp. (Fig. 11) have a characteristic leaf-shaped, broad, flattened body. Externally, they are very similar to *Obama* sp. 6 *sensu* Carbayo et al. (2013) from Brazil (F Carbayo, pers. comm., 2013). One specimen from Bordils locality (Loc code V in Table 1) is deposited at the NHMUK (**NHMUK 2014.5.13.8**).

### Phylogenetic results

We inferred ML trees to check the diagnosis of the introduced specimens and to determine their level of relatedness to the ones from the original areas of distribution. For this reason, the datasets included, when possible, sequences belonging to morphologically diagnosed specimens from the original area of distribution of the putative introduced species (obtained for this study or coming from GenBank; Table 2).

**Figure 12:**
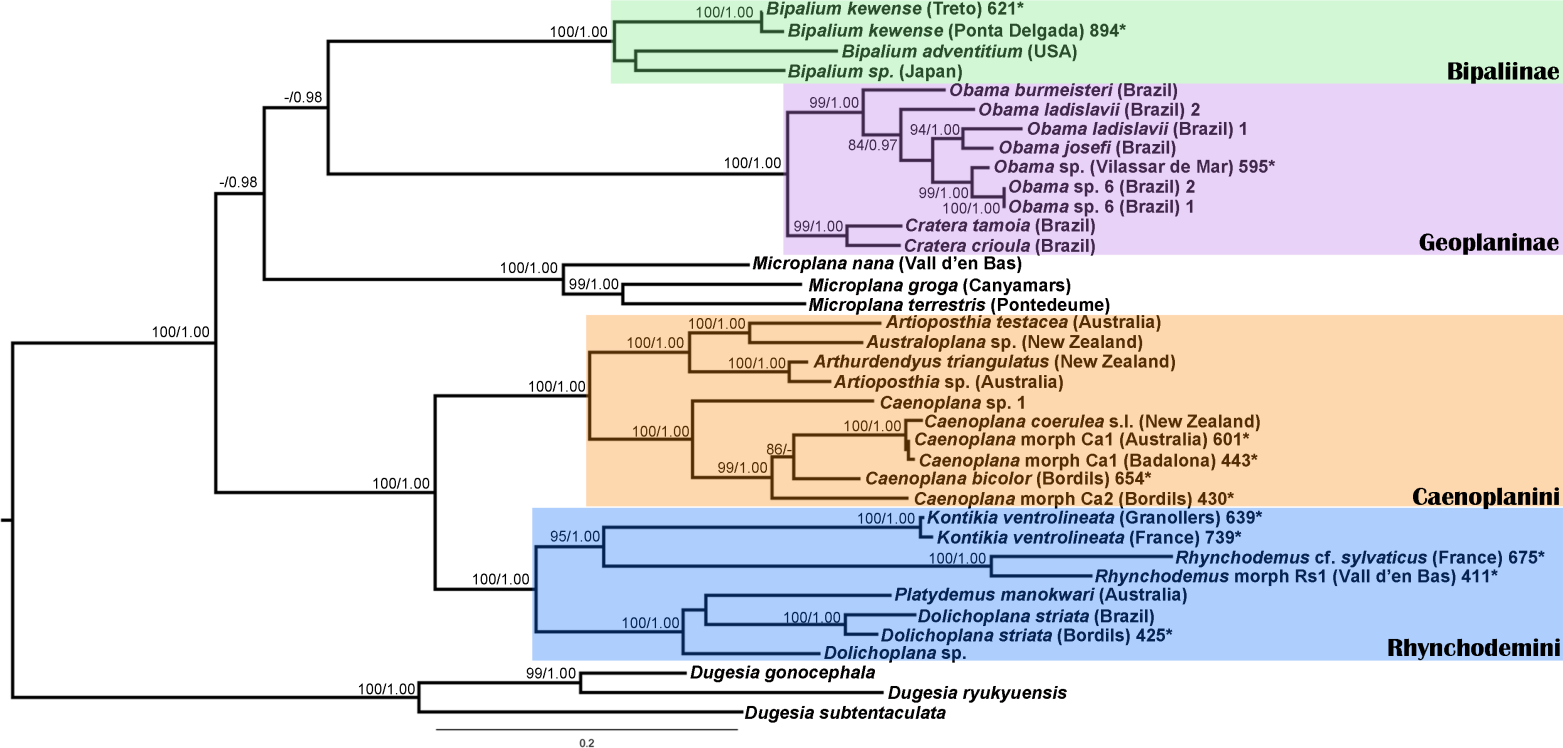
Maximum likelihood (ML) tree of the Geoplanidae subfamilies and tribes (Bipaliinae, Geoplaninae, Caenoplanini, and Rhynchodemini). Tree inferred from the concatenated dataset (Cox1 and 28S genes). Three *Dugesia* species as outgroups. Values at nodes correspond to bootstrap (>75) for ML and posterior probability (PP) values from the Bayesian analysis (>0.95).

**Figure 13:**
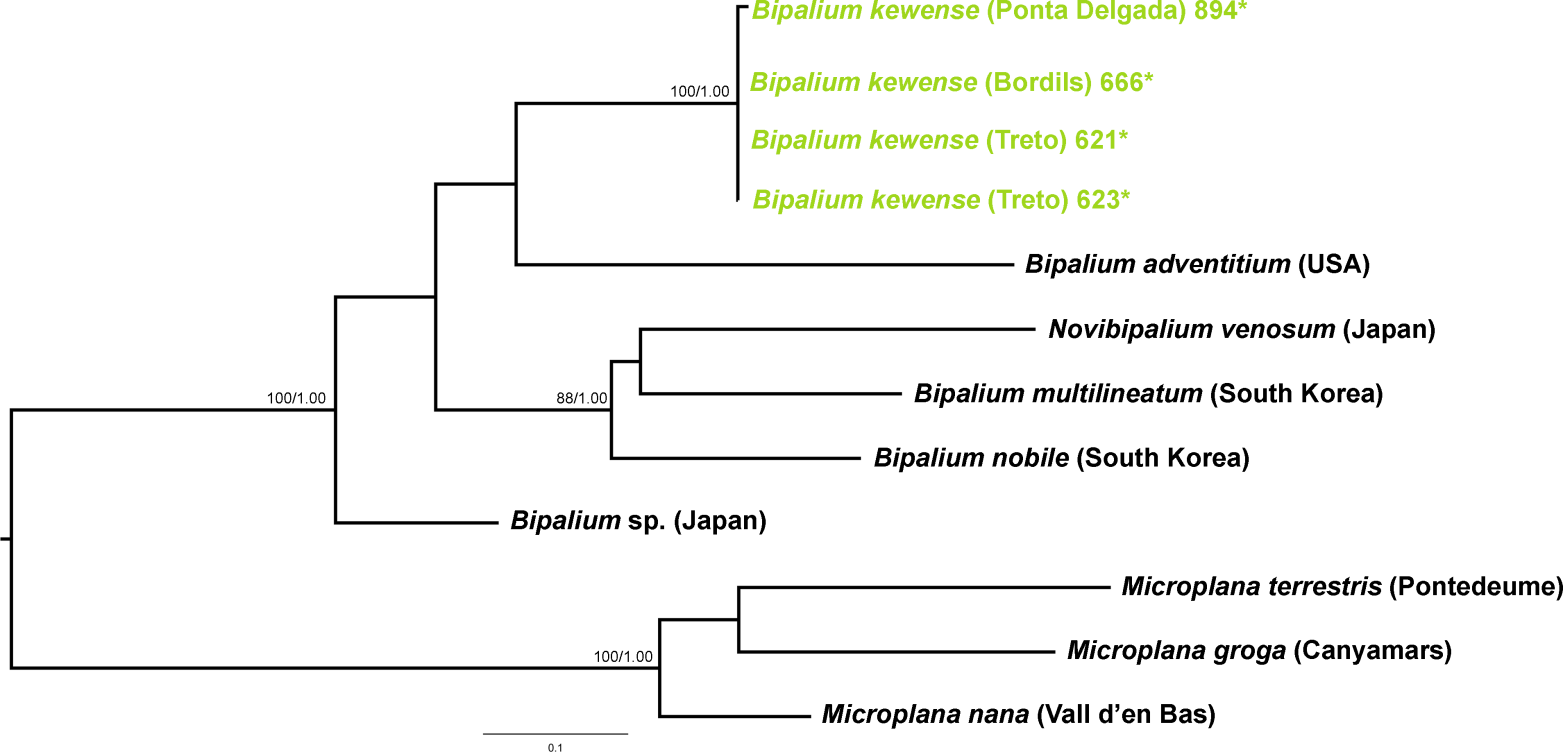
Bipaliinae dataset ML tree. Tree inferred from the Cox1 gene. Three *Microplana* species as outgroups. Values at nodes correspond to bootstrap (>75) and PP (>0.95) values.

**Figure 14:**
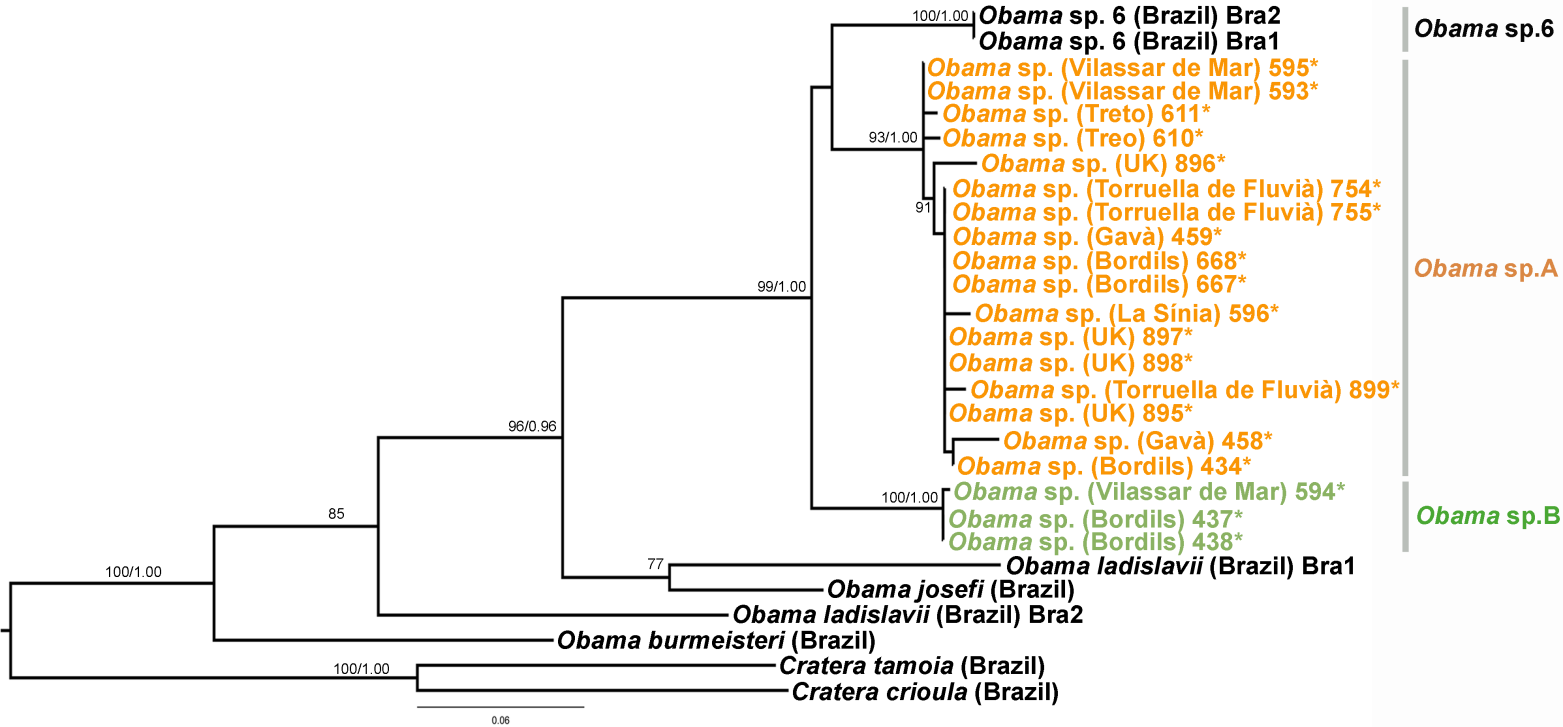
Geoplaninae dataset ML tree. Tree inferred from the Cox1 gene. Two *Cratera* species as outgroups. Values at nodes correspond to bootstrap (>75) and PP (>0.95) values.

**Figure 15:**
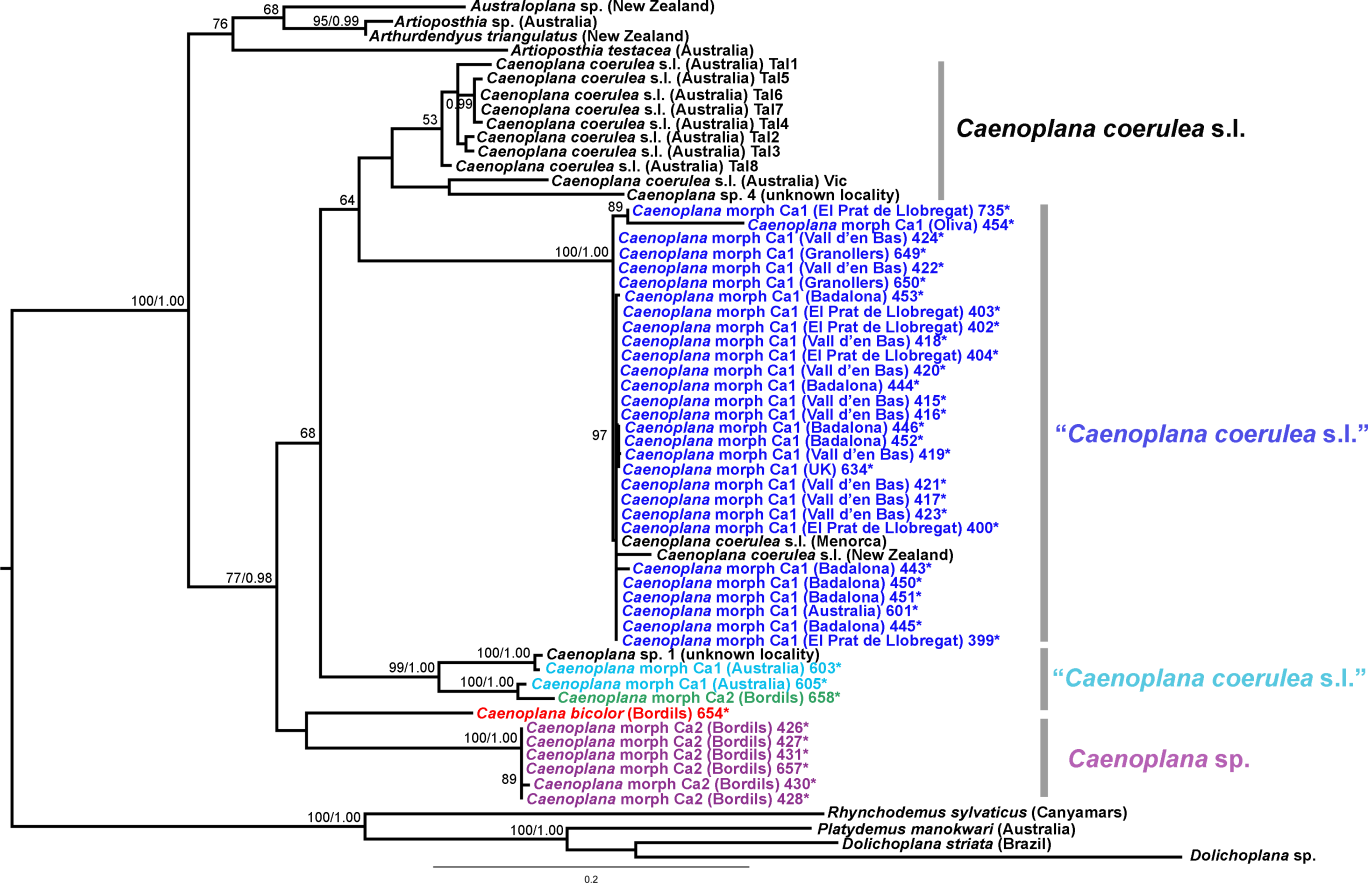
Caenoplanini dataset ML tree. Tree inferred from the Cox1 gene. One *Rhynchodemus* species, one *Platydemus* species, and two *Dolichoplana* species as outgroups. Values at nodes correspond to bootstrap (>75) and PP (>0.95) values.

**Figure 16:**
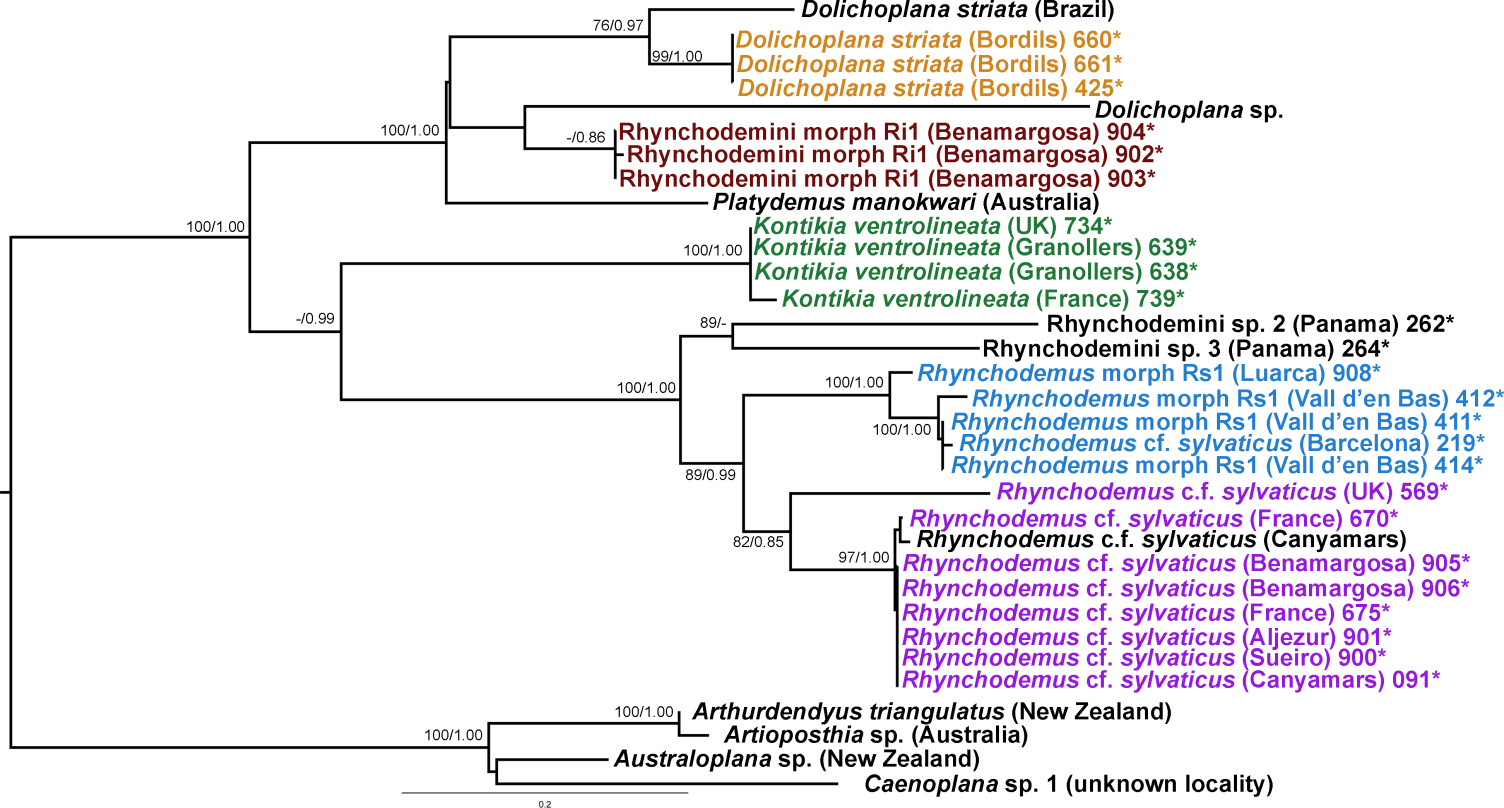
Rhynchodemini dataset ML tree. Tree inferred from the Cox1 gene. One species of *genres Arthurdendyus*, *Artioposthia*, *Australoplana* and *Caenopolana* as outgroups. Values at nodes correspond to bootstrap (>75) and PP (>0.95) values.

We obtained 28S sequences for 15 individuals. One or two sequences from each morphotype were aligned together with 19 GenBank ingroup sequences and 3 outgroup sequences belonging to the *Dugesia* genus (terrestrial planarians sister group; Carranza et al., 1998; Álvarez-Presas, Baguñà & Riutort, 2008). Cox1 sequences were obtained for all individuals included in the study (Table 2). To obtain a more detailed picture of the situation within the main clades, including introduced planarians found on the concatenated analysis, we split the Cox1 sequences into four new datasets, one for each subfamily, tribe or genus: Caenoplanini (56 ingroup + 4 outgroup), Geoplaninae (26 ingroup + 2 outgroup), Bipaliinae (9 ingroup + 3 outgroup) and Rhynchodemini (29 ingroup + 4 outgroup). For each clade, its sister group was selected as the outgroup as shown on the concatenated analysis and/or previous studies (Álvarez-Presas, Baguñà & Riutort, 2008). The best-fit model of sequence evolution for the 28S was GTR + G and for Cox1 was GTR + I + G. We inferred a ML tree with partitions from a concatenated dataset including 37 individuals for which both 28S and Cox1 sequences had been obtained (Fig. 12). The ML trees obtained from the Cox1 datasets are shown in Figs. 13–16.

For the concatenated dataset, the ML tree showed most introduced specimens constitute monophyletic groups together with representatives of their species coming from the original distribution area or other introduced localities. We have found introduced planarians in the IP for all non-autochthonous terrestrial planarians subfamilies; in the case of the Rhynchodeminae there are even representatives from two tribes (Rhynchodemini and Caenoplanini).

Within the Bipaliinae, *Bipalium* specimens found in the IP constitute a monophyletic group together with *Bipalium* sequences from other species, *B. adventitium* being the closest relative in the Cox1 tree (Figs. 12 and 13). The genetic diversity among the four *B. kewense* sequences, coming from the IP and Açores Islands, was very small.

In the Geoplaninae clade (Figs. 12 and 14) the introduced specimens found in the IP constitute a monophyletic group with a still not-described species from Brazil (*Obama* sp. 6 after Carbayo et al., 2013, Fig. 14). In the Cox1 tree, specimens coming from the IP, United Kingdom (both introduced) and Brazil (original area) constitute a highly-supported monophyletic group. Within this group, the introduced individuals are divided in two quite differentiated clades (*Obama* sp.A and *Obama* sp.B in Fig. 14), also distinctly separated from the Brazilian individuals. All the UK individuals fall within the clade *Obama* sp.A.

The Caenoplanini clade (Figs. 12 and 15) includes a high number of introduced individuals and the broadest diversity of sequences. Even *Caenoplana coerulea* sequences, either coming from GenBank, or from the individuals sent by our collaborator in Australia, are found in very distinct genetic clades pointing to the existence of more than one species (see Discussion). For this reason, we use the name *Caenoplana coerulea* s.l. to refer to all those specimens. In the concatenated tree, the representative of *Caenoplana* morphotype Ca1 is closely related to *Caenoplana coerulea* s.l. from Australia, while *Caenoplana* morphotype Ca2 is the sister group of a clade constituted by *C. coerulea* s.l. and *C. bicolor*. The divergence among these three lineages can be appreciated when compared to the other subfamilies present in the tree. In the Cox1 tree (Fig. 15) genus, *Caenoplana* again shows high levels of genetic diversity, evidenced by the long branches separating its subclades. Most *Caenoplana* morphotype Ca1 from the IP constitute a low diversity clade including *C. coerulea* s.l. from its original area (Australia) and also from UK and Menorca (also introduced). This clade is sister to another group including *C. coerulea* s.l. originally from different localities in Australia (Sunnucks et al., 2006); however, the differentiation among these two clades is extremely high. The other two *Caenoplana* morphotype Ca1 individuals, coming from Townsville (Australia), constitute a highly differentiated clade that also includes a GenBank sequence identified only to the genus level and one of the introduced individuals. Finally, there is a clade including only introduced animals, one of them identified as *C. bicolor* and the rest as morphotype Ca2. The genetic differentiation between the two lineages within this clade is nonetheless extremely high.

In the Rhyncodemini clade (Figs. 12 and 16) we find representatives of three genera in the IP. *Dolichoplana striata* sequences form a monophyletic clade in the Cox1 tree, including three introduced animals in the IP and one coming from Brazil. The individuals assigned to Rhynchodemini morphotype Ri1 collected in Málaga (Spain, Loc code G in Table 1; Vila-Farré et al., 2011) cannot be assigned to any species, although they probably belong to *Dolichoplana* given the relationships they show in the Cox1 tree. The four *K. ventrolineata* specimens analyzed constitute a monophyletic group with a low variability, the French representative being the more divergent. The genus *Rhynchodemus* is represented by at least three species in the Cox1 tree. *Rhynchodemus sylvaticus* (considered an European autochthonous species), *Rhynchodemus* morphotype Rs1, and a clade including two individuals from Panamá that we had ascribed to the Rhynchodemini by their external appearance, and they appear likely to belong to the genus *Rhynchodemus*. It should be noted that the specific identification of all *R. sylvaticus* specimens found in the IP (Boix & Sala, 2001; Mateos et al., 2009; Vila-Farré et al., 2008; Vila-Farré et al., 2011) have been made based exclusively on external morphology (for this reason all these specimens have been considered *Rhynchodemus* cf. *sylvaticus*). *Rhynchodemus* cf. *sylvaticus* clade, including representatives from Spain, Portugal, UK and France, together with a specimen identified in a previous study (*Rhynchodemus* cf. *sylvaticus* (Canyamars)) is a sister group of a clade constituted by *Rhynchodemus* morphotype Rs1 and one specimen of *R*. cf. *sylvaticus* (specimen 219).

### Specimen distribution

Figure 3 and Tables 1 and 2 show the sampling localities of the animals analyzed in this study. In all the plant nurseries, only one species of terrestrial planarian was found (*Bipalium kewense,* Rhynchodemini Ri1 or *Obama* sp.), except in Bordils where six species were found (Table 1, Loc-code V). The rest of the localities also contained a single species, with the exception of Treto (a garden, Loc-code U) with two species, and the two “semi natural areas” situated in Vall d’en Bas (Loc-code P) and in Granollers (Loc-code M) also with two species each. *Obama* sp. was the species most frequently found in plant nurseries, while *B. kewense* predominated in private gardens. In the semi natural areas only the species *K. ventrolineata*, *C. coerulea* s.l., and *Rhynchodemus* Rs1 (not found anywhere else) have been found.

### Potential species distribution modelling

**Figure 17:**
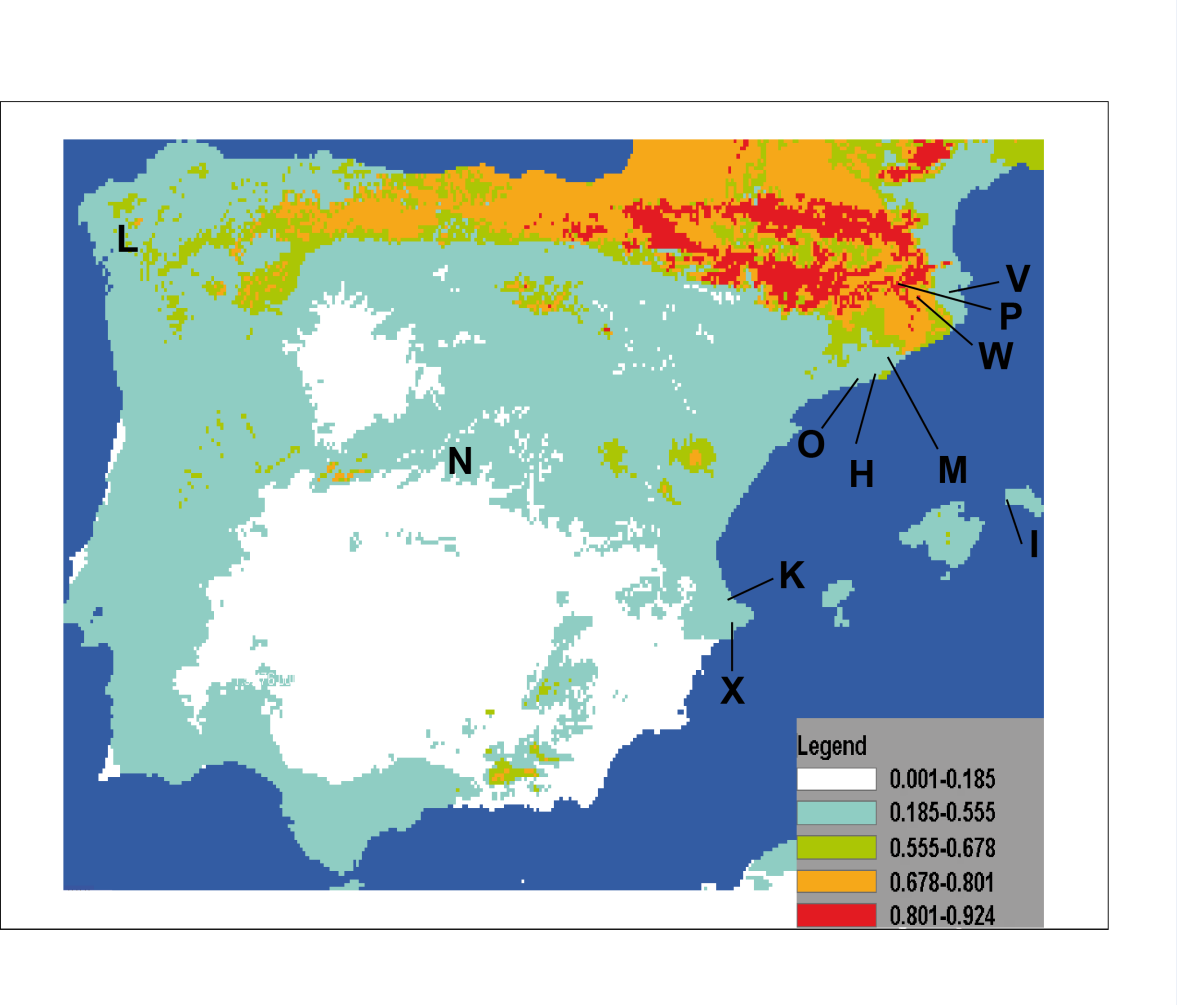
Potential distribution of *Caenoplana coerulea* species across the Iberian Peninsula. The color gradient indicates the predicted likelihood that the environmental conditions suitable for the species based on the MaxEnt average output. Letters indicate localities where *C. coerulea* has been found, locality codes correspond to those in Table 1.

The result of projecting models for the potential distribution of *C. coerulea* s.l. in the IP presents mean values of AUC beyond 0.9 (0.974) and significance for all tests of omission, which indicates good performance of the models. Furthermore, predictions were significantly different from random because binomial omission test thresholds were significant (*p* < 0.01) in all 1,000 runs. A composite map showing the potential distribution models for *C. coerulea* s.l. species projected on current climate layers is provided in Fig. 17.

The results of the potential distribution of the species in the IP, based on data from its current distribution in their region of origin (Australia), show that the species can find extremely suitable areas for its survival and expansion is the northern region, where the appropriate temperature and humidity conditions occur.

## Discussion

### Species identification, or, how many species are out there?

External morphology (Figs. 4–14), analysis of histological sections, and phylogenetic inference from molecular data (Figs. 12–16) have revealed the presence of five clearly identifiable species of introduced exotic land planarians in the IP: *Bipalium kewense* (Bipaliinae), *Caenoplana bicolor*, *Caenoplana coerulea* s.l. (Ca1), *Dolichoplana striata* (Rhynchodeminae, Rhynchodemini), and *Kontikia ventrolineata* (Rhynchodeminae, Caenoplanini). However, the phylogenetic trees obtained and the analysis of the external appearance of the specimens indicate that probably at least five more species were present, including Rhynchodemini morph Ri1, *Rhynchodemus* morph Rs1, *Obama* sp. and two more species within *Caenoplana*:*Caenoplana* morph Ca2 and probably some individuals of *Caenoplana* morph Ca1 (see below).

The assignation of *Bipalium kewense* is based on its characteristic external morphology (see Hill & Merickel, 2011; Jones, 1998). There are no published Cox1 gene sequences for this species in Genbank, so those presented in this paper are the first available. Phylogenetic analysis of these sequences point to an introduction from the same lineage. Surprisingly, all sequences belonging to *Kontikia ventrolineata* (coming from Spain, France and UK) are situated within the Rhynchodemini clade with high support in both trees. This situation contradicts the taxonomy proposed by Sluys et al. (2009) where the genus *Kontikia* belongs to tribe Caenoplanini.

The genetic differentiation observed within the group constituted by the genus *Caenoplana*, monophyletic in the trees, leads us to predict that it includes more than one species. In the Cox1 tree (Fig. 15), at least three monophyletic groups seem to be clearly defined and probably represent different species. In fact, *C. coerulea* is considered by a specialist in this group (L Winsor, pers. comm., 2013) as a complex of species, on the basis of internal anatomical characters and stripe morphology. According to Winsor, there are at least three species that are distinguishable morphologically; but there are probably more than three species in the area of origin. One of the problems with the group is that the type of the species is non-sexually mature. Hence, to clarify the situation and number of the species in this group, a broad sampling in its original area of distribution is required, followed by a thorough morphological and molecular study. Nonetheless, for the purpose of the present paper, the evidence is clear that at least three different genetic lineages from Australia have been introduced in the IP, probably independently.

In the case of *Rhynchodemus* Rs1, we cannot be sure if this is a distinct species or simply a differentiated lineage of *R.* cf. *sylvaticus*. The latter has been generally regarded as an introduced species in Europe from USA (Jones, 1988), but it is also considered as probable species native to Europe (Jones, 1998; Jones, 2005) and introduced in the USA from Europe (Ogren & Kawakatsu, 1998). The type locality of *R. sylvaticus* is Philadelphia, Pennsylvania, USA (1851). This species has a wide distribution in the IP and two of the locations are plant nurseries, one in Barcelona (Vila-Farré et al., 2008) and one in Málaga (Vila-Farré et al., 2011), while the other localities can be considered natural habitats. In our molecular analysis there was no separation of specimens according to their locality type (natural or artificial). Two distinct clades of European *Rhynchodemus* were obtained (Fig. 16), suggesting the existence of two different species with similar external morphology.

In the case of Rhyncodemini Ri1, this species probably belongs to the genus *Dolichoplana*; however, we were only able to obtain three specimens and none of them were sexually mature.

When specimens of *Obama* sp. were first found in the UK and the IP, they were identified as *O. marmorata* (Schultze & Müller, 1857) due to their external appearance; however, molecular data (M Riutort, unpublished data, 2014) showed that the European specimens did not constitute a monophyletic group with that species, indicating that they belonged to an unknown, still undescribed, Geoplaninae. Sampling performed in Brazil since then has found another species (*Obama* sp.6), which is also externally very similar. Molecular data show that it is closely related to the individuals found in Europe (M Riutort, unpublished data, 2014). As in the previous case, a morphological and molecular study should be undertaken to clearly delimit and describe the new species. The two clades found in our Cox1 tree (Fig. 14), that may represent two different species, suggest that there have been two independent introductions into the IP from different native sites in Brazil.

Overall, we have shown that at least ten introductions have occurred in the IP. These introductions include species from all the non-European terrestrial planarian subfamilies from native localities as far as South America and Australia. Since most of these species have previously been reported to have been introduced in other countries, the introductions into the IP have probably not been directly from the source countries, but were more likely to be indirect, following plant trade routes. In most cases, all the individuals from the same species found in the different localities are nearly identical, even when compared between Spain and the UK, which can be interpreted as the result of a single introduction (or a single exportation from the place of origin). In others, as in the case of *Caenoplana*, the observed diversity clearly indicates that the introductions were from different lineages within this group and is likely to be the consequence of more than one export from the native area.

### What makes terrestrial planarians so successful as introduced species?

Temperature, humidity and food availability are the three basic factors determining the geographical distribution of terrestrial planarians (Boag, Yeates & Johns, 1998). The feeding habits of the introduced species in the IP indicate that all of them feed on invertebrate soil fauna (Table 3). In plant nurseries and greenhouses microclimatic conditions are maintained artificially (high humidity and stable temperature) and are likely to favor the presence of stable populations of many species of terrestrial invertebrates. In nurseries we visited, especially under flowerpots, we have observed the presence of numerous specimens of snails, slugs, earthworms, millipedes, isopods, beetles and various groups of microarthropods, including springtails. Therefore, in this very suitable artificial microhabitat, there is likely to be a greater number of species of terrestrial flatworms (as is the case of Bordils, Loc code V in Table 1, where six species were detected in the same greenhouse).

**Table 3:** Feeding habits of the introduced terrestrial flatworm species in the Iberian Peninula. Native region sensu Winsor, Johns & Barker, 2004.

Species (native region)	Prey	Reference
*Bipalium kewense* (Vietnam to Kampuchea, possibly extending to Malaysia)	Earthworms	See Winsor, Johns & Barker, 2004 for refs
*Caenoplana coerulea* (Eastern Australia)	Gastropods, arthropods, earthworms isopods, diplopods, earwings *Ommatoiulus moreletii* (diplopod) beetles diptera larvae	See Winsor, Johns & Barker, 2004 for refs Olewine, 1972 Terrace & Baker, 1994 Mateos et al., 2013 Barnwell, 1978
*Caenoplana bicolor* (unknown)	Isopods	Observations on captive specimen by HD Jones
*Caenoplana* C02 (unknown)	Unknown	–
*Dolichoplana striata* (Indo-Malay region)	Earthworms	See Winsor, Johns & Barker, 2004 for refs
*Kontikia ventrolineata* (Queensland, Australia)	Gastropods, isopods snails, slugs, hawkmoth caterpillars isopods isopods earthworms	See Winsor, Johns & Barker, 2004 for refs Great Britain Non-Native Species Secretariat, 2013 Froehlich, 1956 Olewine, 1972 Present study
*Obama* (Brazil^*^)	Mollusks, earthworms	F Carbayo (pers. comm.)
Rhynchodemini Ri1 (unknown)	Unknown	–
*Rhynchodemus* Rs1 (unknown)	Unknown	
	For Rhynchodemus [genus]:	
	Springtails	Wallner, 1937
	Springtails	Froehlich, 1956
	Springtails	Ogren, 1985
	Woodlice	Jones, 2005

**Notes.**

* Sensu Carbayo et al., 2013.

Land planarians and their cocoons are very often associated with the soil of plants in pots and certain types of fresh vegetables (Ogren, 1985; Mather & Christensen, 1992; Hogan & Dunne, 1996). The transport of these pots and materials (which can occur over international and intercontinental distances) may permit the transport of associated planarians and/or cocoons, which is the primary means of introduction of exotic species of terrestrial planarians into different contaminated countries (Winsor, Johns & Barker, 2004). The suitable conditions in the plant nurseries and garden centers may explain their introduction success. In recent decades, the adoption of free market policies and trade agreements have reduced barriers to plant trade among different countries (Dehnen-Schmutz et al., 2010), but there has been insufficient attention given to how such structural change in international trade can affect the risk of spread of invasive species (Drew, Anderson & Andow, 2010). Depending on the intricate network of commercial interactions among European countries (see Dehnen-Schmutz et al., 2010), we expect a huge European dispersal of exotic animal species associated with this trade.

### Will planarians become invasive in the Iberian Peninsula as has occurred in other areas?

Exotic species present in an area could be categorized as introduced (detected in the area but with unknown status), adventives or not established (they reproduce occasionally in the area not constituting stable populations), naturalized or established (they form stable reproductive populations in the area) and invasive (established and well spread in the area) (Richardson et al., 2000; Simberloff et al., 2013). The “tens rule” (Williamson & Fitter, 1996; Williamson & Brown, 1986; Williamson, 1996) predicts that just one of hundreds of introduced species becomes invasive (about 10% of the introduced species are established, and that 10% of those become invasive). Based on the premise of the “tens rule”, some researchers minimize the potential impact of exotic species (National Research Council, 2002; Campbell & Gibson, 2001), while others warn that this risk minimization is dangerous and, with respect to the possible impact of introduced species, the adoption of the precautionary principle is crucial (Jarić & Cvijanović, 2012), but unlikely! The problem with this sort of assumption or calculation is that, in most cases, we simply have no knowledge of the unsuccessful introductions.

In the case of terrestrial planarians, some species are very tolerant of habitat modification (Cannon et al., 1999; Carbayo, Leal-Zanchet & Vieira, 2002), facilitating their survival in humanized environments. Many introduced species of terrestrial planarians are found confined to these types of habitats (parks, private gardens, plant nurseries), but it is not known whether this distribution is so restricted due to environmental constraints (planarians, coming from tropical habitats cannot live outside these artificial habitats in the European environment) or to a low velocity of dispersion to natural habitats (Ducey & Noce, 1998). In our case, most specimens occurred in confined areas (gardens and nurseries). However, *Rhynchodemus* Rs1, *C. coerulea* s.l. and *K. ventrolineata* have been also found in recently restored areas that were more or less connected to natural and agricultural environments, which increases the danger of their becoming naturalized or even invasive.

In the particular case of *C. coerulea* s.l., we performed a potential distribution study to check whether the area around its present introduced localities in the IP may be suitable for its expansion. The results show that the potential distribution of the species (Fig. 17) indeed includes the countryside that was nearby to the localities of the IP where it is already present. The most suitable area is the northern IP. This is not surprising when we consider that in this northern region, the climatic conditions (temperature and humidity) are also more optimal for the presence of native land planarians (Mateos et al., 2009; Álvarez-Presas et al., 2012). Thus, we show that by having suitable climatic databases, it is possible to model the potential distribution of introduced species, and thus predict their risk of becoming invasive. If we add to this information the knowledge of some biological features of the terrestrial planarian species, such as their prey preferences, we may be able to make an even more precise image of the sites where it is more likely for the species to become invasive and thus concentrate prevention efforts in those areas.

Our results show that *C. coerulea* s.l. is apparently the most successful colonizer, since it is the only species present in all three unconfined (semi natural) areas sampled. This may be because it feeds on several groups of arthropods that are abundant in areas where this species has been detected (isopods, beetles, diplopods). The three species (*Rhynchodemus* Rs1, *C. coerulea* s.l. and *K. ventrolineata*) we find in unconfined environments feed on arthropods, whereas the other species (found only in confined environments) do not feed on arthropods, but instead on other invertebrates that require extremely wet habitats. Hence, land planarian species that feed on arthropods have their food “secured” in environments with a Mediterranean climate and, as a consequence, have a higher likelihood of being successful and becoming established or even invasive.

### What consequences might the introduction of flatworms have on human economies and biodiversity?

Another important question is: what are the negative effects of the spread of these species? In literature, the primary problems reported are related to economic consequences for agricultural activities (Boag & Neilson, 2006; Boag, Neilson & Jones, 2010). As predators of earthworms, planarians can cause soil drainage and fertility to be severely compromised. The ecological consequences of the presence of these predators depends on their propagation speed and efficiency, but could have significant effects on processes mediated by earthworms in both agroecosystems and forests (Lilleskov et al., 2010). Although there is still no direct impact study of the presence of invasive planarians on agricultural production (Boag, Neilson & Jones, 2010), data from farmers with infested farmland and from the scientific literature have suggested that it could reduce grass yields significantly (Boag & Neilson, 2006).

No reference has been made to the effect of these species on autochthonous populations of terrestrial planarians, probably because the knowledge of the autochthonous fauna is very scarce. In the IP we have already performed some studies on the autochthonous terrestrial planarian fauna and found that it is very diverse, including at least 15 species, of which some contain a great deal of genetic diversity (Mateos et al., 2009; Álvarez-Presas et al., 2012). The potential arrival of some of these introduced species in natural habitats, where the autochthonous ones are localized (as predicted by the potential distribution studies), would have very negative consequences. Since exotic planarians are, in general, bigger in size, more voracious, have more aggressive behavior, and sometimes appear to have a generalist diet (pers. obs.), they may be more resistant to extreme conditions than the native species.

### A cautionary tale: plant trading and landscape restoration

An important question raised by all these observations is whether governments in Europe should be asked to propose new, more restrictive rules on the trade of plants coming from outside, or alternatively, to establish better controls or protocols to avoid the introduction of unwanted organisms together with the plants. However, it is probably now too late to have an impact on the transport of species around the world. Nonetheless, we are still in time to avoid invasions of terrestrial planarians. The restoration of degraded areas involves planting native plant species. These plants are available from nurseries and transported to the restoration areas accompanied by a certain amount of soil on the roots. If this land is not subject to any preventive treatment, it may be contaminated with organisms that are also introduced in the area that is being restored. Among these organisms may be unwanted species that, if given the right conditions, can become invasive. It is important to warn agencies conducting such restorations of these dangers and ask stakeholders to include in the protocols of landscape restoration the necessary steps to avoid these unwanted introductions.

Some simple, easy-to-perform sanitizing procedures, such as heating the soil (EPPO, 2000a; EPPO, 2000b; SEERAD, 2000; Sugiura, 2008) before transplanting the nursery plants to the natural environment, may be sufficiently effective and reliable to ensure that there is no concomitant dispersal of flatworms. Such procedures, together with a periodic analysis of the introduced species present in garden centers and nurseries, and a study of the potential areas of flatworm distribution, would also help avoid the introduction of terrestrial planarians into areas where they are more likely to become invasive (DEFRA, 2005; DOVE, 2012).

## Additional Information and Declarations

### DNA Deposition

The following information was supplied regarding the deposition of DNA sequences: GenBank. See Table 2.
